# RNA-binding proteins involved in post-transcriptional regulation in bacteria

**DOI:** 10.3389/fmicb.2015.00141

**Published:** 2015-03-03

**Authors:** Elke Van Assche, Sandra Van Puyvelde, Jos Vanderleyden, Hans P. Steenackers

**Affiliations:** Centre of Microbial and Plant Genetics, Department of Molecular and Microbial Systems, KU LeuvenLeuven, Belgium*

**Keywords:** post-transcriptional regulation, RNA-binding proteins, bacteria, working mechanisms, biotechnological applications, regulation of translation, stability regulation

## Abstract

Post-transcriptional regulation is a very important mechanism to control gene expression in changing environments. In the past decade, a lot of interest has been directed toward the role of small RNAs (sRNAs) in bacterial post-transcriptional regulation. However, sRNAs are not the only molecules controlling gene expression at this level, RNA-binding proteins (RBPs) play an important role as well. CsrA and Hfq are the two best studied bacterial proteins of this type, but recently, additional proteins involved in post-transcriptional control have been identified. This review focuses on the general working mechanisms of post-transcriptionally active RBPs, which include (i) adaptation of the susceptibility of mRNAs and sRNAs to RNases, (ii) modulating the accessibility of the ribosome binding site of mRNAs, (iii) recruiting and assisting in the interaction of mRNAs with other molecules and (iv) regulating transcription terminator/antiterminator formation, and gives an overview of both the well-studied and the newly identified proteins that are involved in post-transcriptional regulatory processes. Additionally, the post-transcriptional mechanisms by which the expression or the activity of these proteins is regulated, are described. For many of the newly identified proteins, however, mechanistic questions remain. Most likely, more post-transcriptionally active proteins will be identified in the future.

## INTRODUCTION

Bacteria need to survive in constantly changing environments. Therefore, they must be able to alter their gene expression in response to environmental signals, causing protein levels to be adjusted according to the needs of the cell. This can be achieved by adjusting transcription initiation with sigma factors and proteins that activate or repress transcription. However, gene expression regulation also occurs after transcription is initiated (Perez-Rueda and Martinez-Nuñez, 2012). The importance of these post-transcriptional regulatory processes is highlighted by the weak correlation that has been observed between RNA and protein abundance (Picard et al., 2009).

Prokaryotic post-transcriptional regulators typically modulate RNA decay, translation initiation efficiency or transcript elongation. Different types of prokaryotic post-transcriptional regulators have been identified, including small RNAs (sRNAs) and RNA-binding proteins (RBPs). sRNAs are typically defined as non-coding RNA molecules that bind with limited complementarity near the ribosome binding site (RBS) of their target mRNA, causing competition with the ribosome for binding to this region. However, the number of sRNAs that deviate from this general definition is increasing (Liu and Camilli, 2010; Storz et al., 2011). The new insights into the post-transcriptional mechanisms of sRNAs and their role in gene expression regulation were reviewed recently (Desnoyers et al., 2013). Here, RBPs involved in post-transcriptional regulation are discussed. For some of these proteins, the mechanism of action and the targets are well described, as for CsrA and Hfq. Their post-transcriptional function in *Escherichia coli* was already reported almost 20 years ago (Liu et al., 1995; Muﬄer et al., 1996). Lately, more insight was gained into the diverse mechanisms these two well-studied proteins use to regulate the expression of their target genes and how they regulate their own expression or activity in *E. coli* and in other bacteria. Additional RBPs involved in post-transcriptional regulation have been identified only recently and not much is known about their post-transcriptional function. In this review, the general working mechanisms of RBPs are discussed first. Afterward, examples of well-known and recently identified proteins, from *E. coli* and from other bacteria, are described.

## GENERAL MECHANISMS OF REGULATORY PROTEINS THAT ACT POST-TRANSCRIPTIONALLY

Bacterial post-transcriptionally active regulatory proteins typically bind RNA molecules and regulate translation initiation, stability, and transcript elongation of their RNA targets, using different regulatory mechanisms. These mechanisms include (i) adaptation of the susceptibility of the target RNAs to RNases, (ii) modulation of the accessibility of the RBS of mRNA targets for ribosome binding, (iii) acting as a chaperone for the interaction of the RNA target with other effector molecules, and (iv) modulation of transcription terminator/antiterminator structure formation, and will be described hereafter.

### ADAPTATION OF THE SUSCEPTIBILITY TO RNases

**FIGURE 1:**
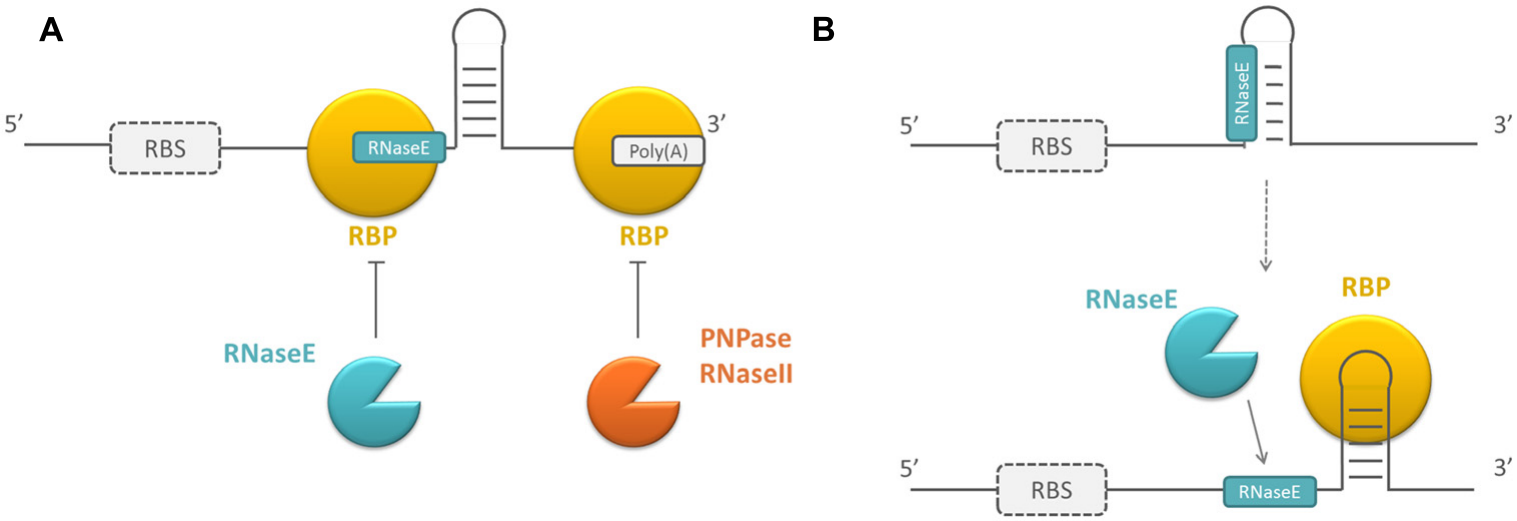
**Schematic overview of the working mechanism of RNA-binding proteins (RBPs) influencing RNase susceptibility. (A)** By directly blocking RNase recognition sites or **(B)** by changing the secondary structure of the mRNAs they bind. Because the targets of RBPs can be both mRNAs and small RNAs (sRNAs) and sRNAs are not translated, the ribosome binding site (RBS) is surrounded by a dotted line. RBPs are depicted in yellow, endoribonucleases in blue, exoribonucleases in orange.

Regulation of RNA stability is an important mechanism to post-transcriptionally control gene expression, as it affects the number of mRNAs that can be translated or the number of sRNAs that can execute their regulatory function. RNA stability is determined by intrinsic RNA elements, such as primary sequence and secondary structure, but can be affected by sRNAs or proteins that bind to the RNA molecule. These proteins are mainly ribonucleases (RNases). In *E. coli*, single-stranded RNA-specific endoribonucleases (e.g., RNaseE and RNaseG) or double-stranded RNA-specific endoribonucleases (e.g., RNaseIII) generally initiate mRNA decay by making endoribonucleolytic cleavages. This yields smaller products that are further degraded by a combination of endo- and exonucleases, like PNPase (polynucleotide phosphorylase), RNaseII, or occasionally RNaseR (reviewed in Kaberdin et al., 2011). sRNAs are mainly degraded by RNaseE and PNPase, or by RNaseIII if the sRNA is hybridized to an mRNA target (reviewed in Saramago et al., 2014). In addition to RNases, other RBPs can play a role in the regulation of RNA stability by modulating the susceptibility of mRNAs and sRNAs to these RNases. Regulatory RBPs can act by directly shielding the recognition sites of RNases involved in the decay of RNA molecules if they have a shared binding preference, e.g., proteins that bind to single stranded AU-rich regions which are also recognized by RNaseE in *E. coli* (Moll et al., 2003). Other regulatory RBPs are involved in the regulation of RNA stability and induce a change in the secondary structure of their mRNA targets upon binding. Consequently, RNase recognition sites become buried or more exposed in locally formed structures, which positively or negatively affects the RNA stability of these molecules, respectively (Barria et al., 2013; see **Figure 1**).

Although RNases are the proteins that are mainly involved in RNA degradation, mRNA modifying enzymes can facilitate mRNA turnover as well. Pyrophosphate removal at the 5′ end by RppH (pyrophosphate hydrolase) and addition of a single stranded poly(A) extension at the 3′ end of the mRNA by PAPI [Poly(A) polymerase I] both promote mRNA degradation. Additionally, the exonucleolytic decay of highly structured mRNAs can be facilitated by RhlB, which unwinds RNA structures in an ATP-dependent way (reviewed in Kaberdin et al., 2011). *E. coli* regulatory RBPs can interfere with the poly(A)-assisted decay of mRNA molecules by binding to the poly(A) tail and protecting the mRNA to which it is bound from degradation (Folichon, 2003). Other regulatory RBPs facilitate mRNA degradation by recruiting RNases or RNA modifying enzymes, e.g., PAPI (De Lay et al., 2013). Because these proteins form a platform for the interaction of RNA molecules and proteins, their mechanism of action is assumed to be different and will be described later.

Many components of the mRNA decay machinery, like the RNases RNaseIII, PNPase, RNaseII, and RNaseR as well as the polymerase PAPI are well conserved across the bacterial kingdom (Kaberdin et al., 2011). This is not the case for the major endonucleases RNaseE/G in *E. coli*. However, functional homologs of RNaseE/G were identified, e.g., RNase J1/J2 or RNaseY in *Bacillus subtilis* (Even et al., 2005; Shahbabian et al., 2009). Therefore, it is likely that the regulatory mechanisms identified for regulatory RBPs influencing the susceptibility to degrading or modifying enzymes in *E. coli* are conserved in other bacteria.

### MODULATING RBS ACCESSIBILITY

**FIGURE 2:**
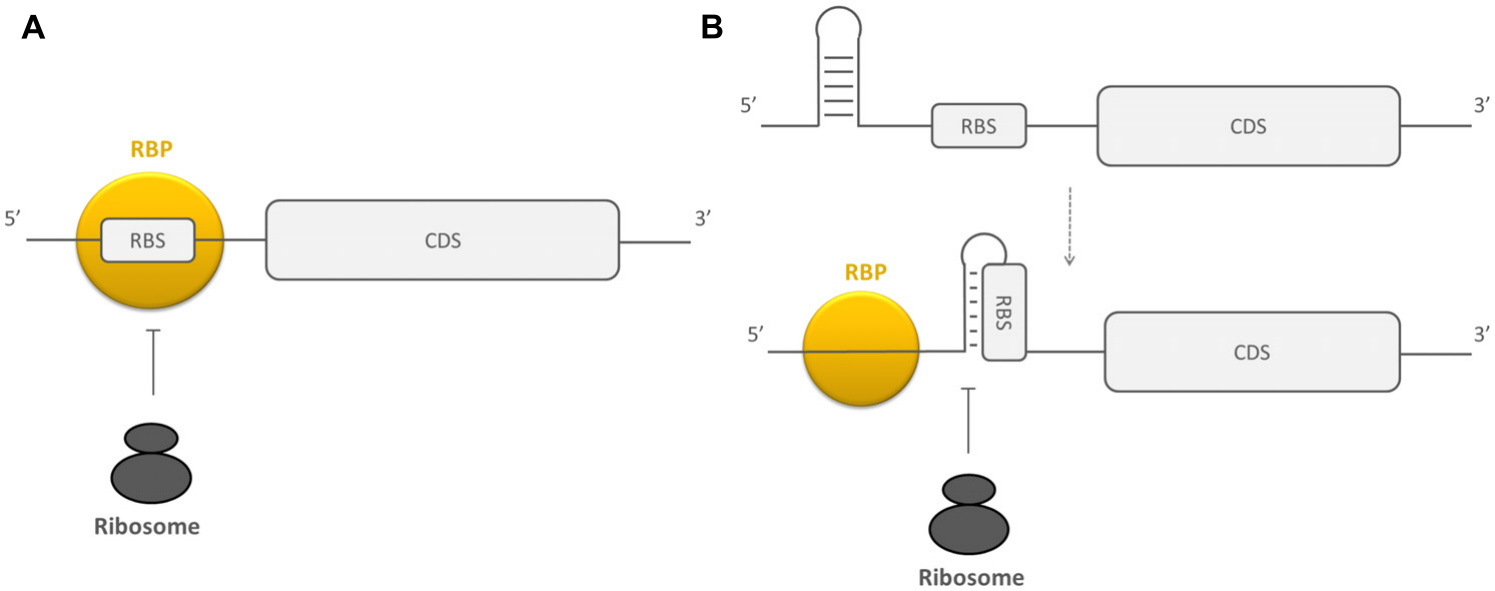
**Schematic overview of the working mechanisms of RBPs influencing the accessibility of the RBS. (A)** By directly blocking this region and **(B)** by changing the secondary structure of the region surrounding the RBS. RBPs are depicted in yellow, the ribosomes in black, CDS, coding sequence.

Besides their involvement in the regulation of RNA stability, RBPs can post-transcriptionally control gene expression by altering the efficiency of translation initiation. Translation initiation of an mRNA requires ribosome binding to the RBS of the mRNA. This RBS contains the Shine–Dalgarno sequence, which is a sequence complementary to the 3′ end of the 16S rRNA. This sequence is important for the recruitment and the correct positioning of the ribosome on the mRNA (Shine and Dalgarno, 1974). The more efficient the pairing between the Shine–Dalgarno region of the mRNA and the 16S rRNA, the more efficient ribosomes are recruited. Although the Shine–Dalgarno region is very important, the interaction region of an initiating ribosome is larger than the Shine–Dalgarno sequence alone and comprises nucleotides -20 to +19 relative to the start codon of mRNAs broadening the region that needs to be accessible in order for ribosome binding to occur (Beyer et al., 1994; Huttenhofer and Fnolier, 1994; Mackay et al., 2011; Desnoyers and Masse, 2012). Regulatory RBPs can modulate the efficiency of translation initiation by directly competing with ribosomes for binding to the ribosome interaction region or by initiating a change in the secondary structure of the mRNA sequence near this region (see **Figure 2**; Dubey et al., 2005; Baker et al., 2007; Irie et al., 2010). The resulting reduction in translation initiation efficiency often causes mRNA stability to be decreased as well. This can be explained by two mechanisms. Firstly, RNaseE can bind internally to a transcript (Mackie, 1998; Kime et al., 2010), but it can also interact with 5′ monophosphorylated transcripts with its 5′ binding pocket (Callaghan et al., 2005). When there is no ribosome bound, the mRNA is not protected from this kind of interaction with RNaseE. Secondly, when translation initiation efficiency is reduced, the spacing of the translating ribosomes on the mRNA is less compact. Consequently, it is more likely that RNase recognition sites in the mRNA become exposed causing transcript decay (Deana and Belasco, 2005).

### RECRUITING AND ASSISTING IN THE INTERACTION WITH OTHER MOLECULES

**FIGURE 3:**
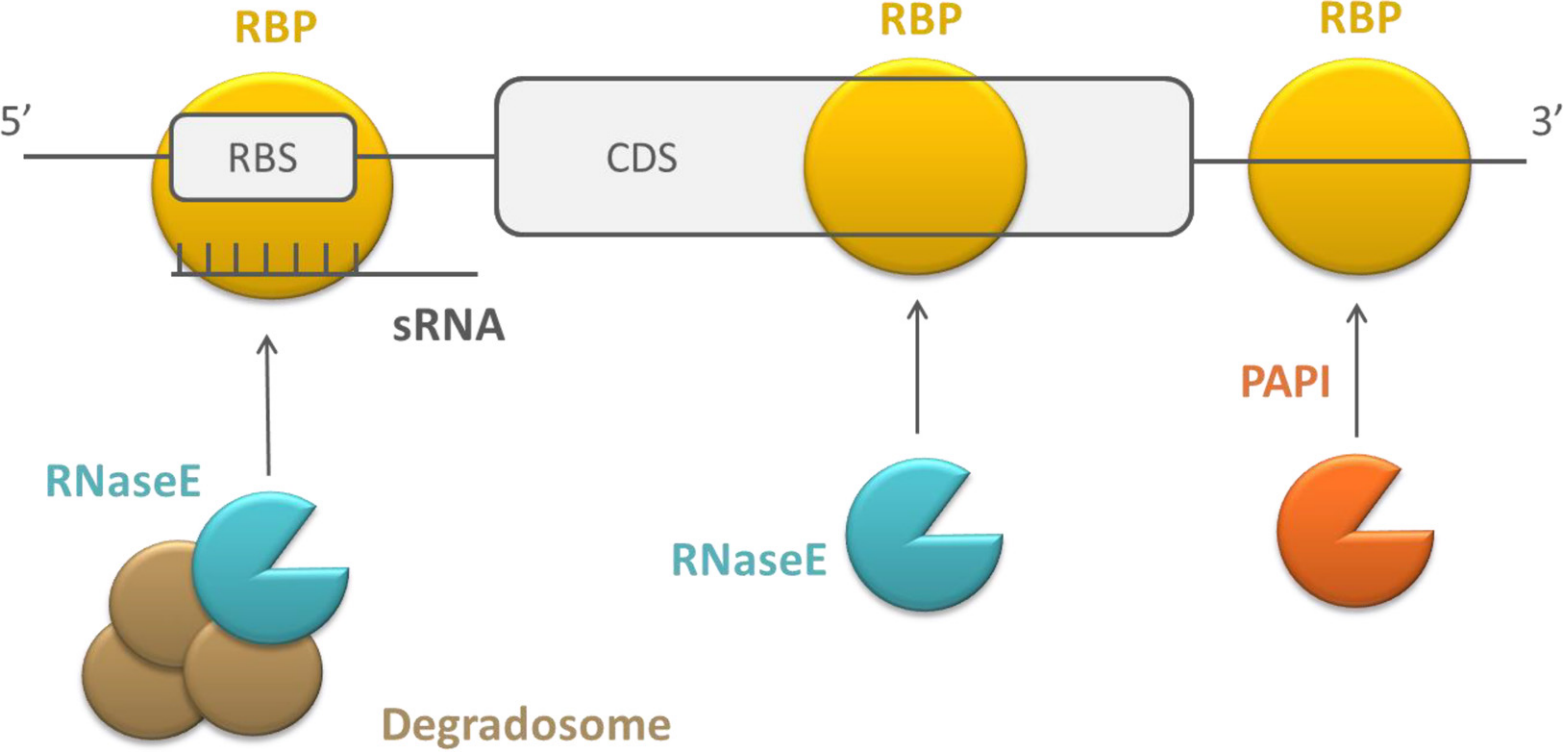
**Schematic overview of the working mechanism of RBPs that recruit and assist in the interaction with sRNAs and proteins.** RBPs are depicted in yellow, endoribonucleases in blue, exoribonucleases in orange, and auxiliary factors of the degradosome in brown, CDS, coding sequence.

RNA stability or translation initiation efficiency can also be affected by RBPs that form a platform to assist in the interaction of other molecules, which consequently affect RNA stability or translation efficiency (see **Figure 3**). The mechanism of action of these RBPs is described here as different compared to the previous two, because they typically bind simultaneously to an RNA target and an effector molecule. The effector molecule bound by the regulatory protein can be an sRNA or a protein. As previously mentioned, sRNAs typically regulate translation efficiency and RNA stability by binding near the RBS of their mRNA targets. Intermolecular basepairing between the sRNAs and mRNAs is facilitated by regulatory RBPs that function as a chaperone (Herschlag, 1995; Soper et al., 2011). The proteins recruited by regulatory RBPs can be proteins facilitating mRNA degradation, e.g., PAPI, RNases, or the degradosome (De Lay et al., 2013). The degradosome is a multiprotein complex in which different components cooperate during mRNA decay. Often, it contains RNaseE as a scaffolding protein and the protein partners PNPase, enolase, and RhlB. However, its assembly is not essential for RNA decay in *E. coli* (Carpousis, 2007). The recruitment of these proteins by other RBPs negatively affects transcript stability.

### MODULATION OF TRANSCRIPTION TERMINATOR/ANTITERMINATOR STRUCTURE FORMATION

**FIGURE 4:**
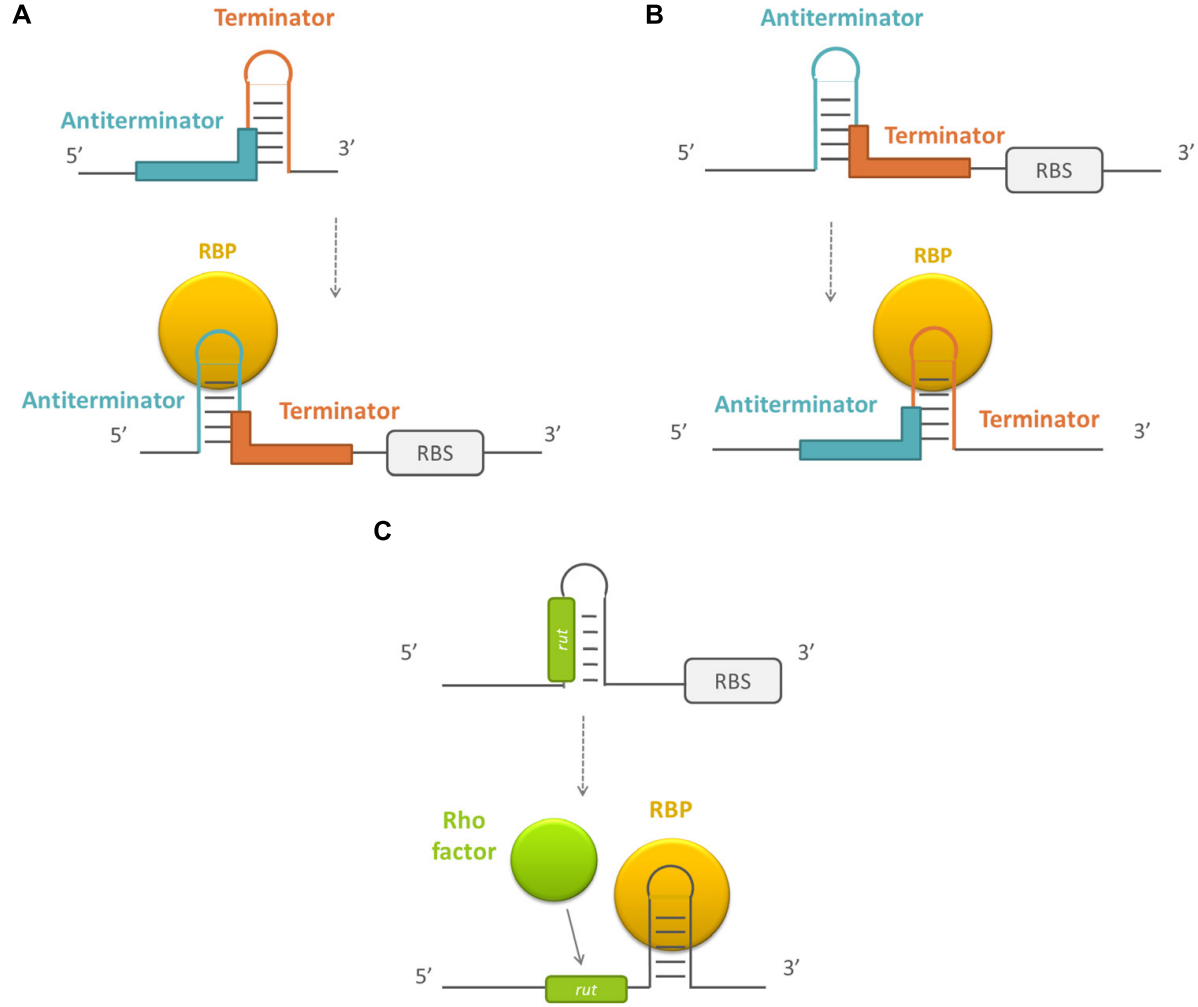
**Schematic overview of the working mechanism of RBPs that modulate transcription terminator/antitermator structure formation. (A)** By stabilizing the antiterminator structure, **(B)** by stabilizing the terminator structure, and **(C)** by exposing a rho utilization site. RBPs are depicted in yellow, the sequences forming the terminator in red, the sequences forming the antiterminator in blue, and the rho utilization sequences (*rut*) en rho factor in green. RBS, ribosome binding site.

A last mechanism by which RBPs can post-transcriptionally affect gene expression is by modulating transcription elongation. After RNA polymerases initiate transcription, transcripts are elongated until a terminator is reached. There are two classes of terminators: intrinsic and factor-dependent terminators. At intrinsic terminators, dissociation of the elongation complex is dependent on the nucleic acid sequence and structure, while factor-dependent termination is dependent on the action of a protein factor, like the Rho-protein (Santangelo and Artsimovitch, 2011). Typically, these terminators are present at the end of the operon. However, some also exist within the 5′ leader region of the transcript. The presence of a terminator at this site prevents transcript elongation to full length. Premature termination can be abrogated by proteins that bind to the polymerase and allow transcription beyond the terminator signals or by the formation of an alternative secondary structure the enables transcription to progress (Stülke, 2002). In the latter process, RBPs can play a role. In the case of intrinsic termination, the RBPs can stabilize either the terminator structure or an alternative secondary structure, the antiterminator, which prevents the terminator form forming. Often the formation of both structures are mutually exclusive (Santangelo and Artsimovitch, 2011; see **Figure 4**). In general, the activity of this type of RBPs is controlled by their phosphorylation state or by a bound ligand, which induces major conformational changes in the proteins (Santangelo and Artsimovitch, 2011), although there are exceptions (Tortosa et al., 1997; Bachem and Stülke, 1998). Furthermore, RBPs can play a role in rho-dependent termination by inducing a secondary structure change, exposing a rho utilization (*rut*) sequence that is normally inaccessible for the rho-factor. Protein binding enables access to this region and rho-dependent transcription termination takes place (Figueroa-Bossi et al., 2014).

## DIFFERENT RNA-BINDING PROTEINS THAT ACT POST-TRANSCRIPTIONALLY IN BACTERIA

A number of proteins involved in post-transcriptional regulation have been identified in *E. coli* as well as in other bacteria. They are listed in **Table 1**. From this list, the global regulatory proteins CsrA and Hfq are best described in literature. CsrA works predominantly by competing with the ribosome for binding to the RBS of its mRNA targets. Hfq is best known for its role in assisting interacting sRNAs and mRNAs, but the protein applies a variety of other mechanisms to post-transcriptionally control gene expression. Besides Hfq and CsrA, there are other proteins that regulate the expression of their mRNA targets using similar working mechanisms. Moreover, additional RBPs have been identified that specifically regulate sRNA stability. For other RBPs affecting translation efficiency or RNA stability, the exact working mechanism is still unknown. Possibly they do use analogous mechanisms compared to those described for the well-known proteins.

**Table 1:** Bacterial RNA-binding proteins (RBPs) that influence gene expression post-transcriptionally.

RBP	Targets	Sequence selectivity	Predominant post-transcriptional working mechanism	Post-transcriptional regulators	Species	Reference
ANTAR containing proteins	Nitrate metabolism and other	Tandem stem-loop	Antitermination, mechanism unclear	/	Widely distributed	Ramesh et al. (2012)
Bgl/Sac family	Carbohydrate utilization genes	Ribonucleotide antiterminator (RAT)	Stabilizing antiterminator	/	Widely distributed	Rutberg (1997)
BpuR	*bpuR* mRNA	/	Inhibiting translation, exact mechanism unclear	/	*Borrelia burgdorferi*	Jutras et al. (2013b)
Csp	Global regulator	Hairpins	Promoting secondary structure change that changes availability for RBS or RNases - Blocking RNase interactions - Stabilizing antiterminator	/	Widely conserved	Barria et al. (2013)
CsrA (RsmA)	Global regulator	RUACARGGAUGU	Competing with ribosome binding - Blocking RNase interactions - Activation of translation by unknown mechanism	small RNAs (sRNAs) CsrB/C (RsmX/Y/Z) FliW protein	Widely conserved	Reviewed in reference Romeo et al. (2013)
CsrD	CsrB/C sRNAs	Unspecific, specificity by accessory proteins	Unclear, possibly by promoting secondary structure change that changes availability for RNases	CsrA protein	Enterobacteriacea	Suzuki et al. (2006)
FbpB	Iron metabolism	/	Unclear, possibly interaction platform sRNA/mRNA - Possibly recruiting RNase and degradosome	/	*Bacillus subtilis*	Gaballa et al. (2008), Smaldone et al. (2012)
FlbT	FliC	/	Unresolved	/	Rhizobiales Caulobacter crescentus	Anderson and Gober (2000), Ferooz et al. (2011)
Hfq	Global regulator	Poly(A)/ARN Poly(U)/AU-rich ssRNA U-rich dsRNA	Interaction platform sRNA/mRNA - Direct blocking of RNase recognition sites - Recruiting PAPI, Crc, RNaseE and degradosome - Competing with ribosome binding	Autoregulation sRNA CrcZ	Widely conserved	Reviewed in reference Sauer (2013)
ProQ	ProP, biofilm	Duplex with ss 5’ and 3’ end	Interaction platform sRNA/mRNA - ribosome association	/	*Escherichia coli*	Chaulk et al. (2011), Sheidy and Zielke (2013)
PyrR	Pyrimidine metabolism	/	Stabilizing anti-antiterminator		*B. subtilis*	Lu et al. (1996)
RapZ	GlmZ sRNA	/	Unclear, possibly recruiting RNaseE or promoting secondary structure change that changes availability for RNases	sRNA GlmY	*E. coli*	Göpel et al. (2013)
RNaseE	General RNA turnover	ss AU-rich region (A/GNAU)	RNase		Widely conserved	Reviewed in reference Mackie (2013)
RodZ	InvE (T3SS) + role in cell shape	/	Unresolved	/	*Shigella sonnei*	Mitobe et al. (2011)
RsmE	Global regulator	RUACARGGAUGU	Direct blocking of RBS	RsmA protein sRNAs RsmY/Z	Pseudomonads	Reimmann et al. (2005)
RsmN/F	Global regulator	RUACARGGAUGU	Direct blocking of RBS	RsmA protein sRNAs RsmY/Z	Pseudomonads	Marden et al. (2013); Morris et al. (2013)
S1	Global regulator	AU-rich ssRNA	Direct blocking of RNase restriction sites	/	Gram-positive bacteria	Hajnsdorf and Boni (2012)
TRAP	Tryptophan metabolism	(NAG)_9-11_	Direct blocking of RBS - Promoting secondary structure change that blocks RBS - Stabilizing terminator	Anti-TRAP protein	*B. subtilis*	Muto et al. (2000), Babitzke (2004), Gollnick et al. (2005)
YbeY	Global regulator sRNAs	/	RNase - Possibly interaction platform sRNA/mRNA	/	Widely conserved	Pandey et al. (2011, 2014)
YopD	T3SS + structural pore component	/	Modifying ribosome	/	*Yersinia* species	Chen and Anderson (2011)

*In the table are the targets and processes in which the regulatory proteins are involved, the sequences they recognize, the working mechanisms they use, the molecules that regulate their activity and the species in which they were identified*.

### CsrA, AN RNA-BINDING PROTEIN PREDOMINANTLY ACTING BY CHANGING RBS ACCESSIBILITY FOR RIBOSOMES

CsrA from *E. coli* and its orthologs in other bacteria, are RBPs that predominantly regulate gene expression by competing with the ribosome for binding to the RBS. CsrA is a widely conserved protein that has been annotated in over 1500 species (Finn et al., 2014). The protein is a global regulator, as illustrated by the changed expression level of approximately 10% of the genes in a *csrA* mutant in different bacteria (Lawhon et al., 2003; Burrowes et al., 2006; Brencic and Lory, 2009). In general, CsrA activates exponential phase functions and represses stationary phase processes (reviewed in Romeo et al., 2013). This is caused by direct and indirect regulatory events, as some CsrA targets encode regulatory proteins themselves (Jonas et al., 2008; Edwards et al., 2011).

To determine the selectivity of the CsrA protein, a SELEX experiment was conducted, enabling the identification of the RNA ligands binding with the highest affinity to CsrA. The CsrA-binding consensus sequence was shown to be RUACARGGAUGU; with the ACA and GGA being 100% conserved. Besides by the presence of this sequence in the mRNA target, CsrA specificity is additionally determined by the secondary structure of the target, as CsrA preferentially binds to RNA molecules that have the binding motif in a hairpin structure, with GGA in the loops of the hairpin (Dubey et al., 2005). The similarity between the CsrA recognition sequence and the consensus sequence of the Shine–Dalgarno region, i.e., AGGAGG, explains why there can be competition between CsrA and the ribosome for binding at this region. However, there are examples of CsrA targets that have CsrA binding sites that do not overlap with the Shine–Dalgarno region. In these cases, one of the CsrA binding sites overlaps with the translation initiation codon (Jonas et al., 2008) or the binding sites are solely present within the coding region (Yakhnin et al., 2011a). In the latter case, CsrA still competes with ribosome binding (Yakhnin et al., 2011a). Generally, the reduced translation initiation efficiency that results from CsrA binding to an mRNA target, leads to mRNA degradation as well (Baker et al., 2002; Dubey et al., 2003; Wang et al., 2005), although there are exceptions (Baker et al., 2007).

While the predominant regulatory mechanism of CsrA is direct competition with the ribosome for RBS binding, the protein can also use other mechanisms. One example is the binding of RsmA, the CsrA ortholog in *Pseudomonas aeruginosa*, to one of its targets, *psl*, which causes a stabilization of a hairpin structure in the region spanning the RBS, blocking the Shine–Dalgarno region and preventing ribosome binding (Irie et al., 2010). Secondly, binding of CsrA can have a positive effect on gene expression by blocking RNaseE interaction sites at the 5′ region of the mRNA of *flhDC* in *E. coli* which has a positive effect on RNA stability (Yakhnin et al., 2014). Thirdly, CsrA has been implicated recently in promoting the translation of the *moaA* mRNA. CsrA binding influences the structure of the *moaA* mRNA, however, this does not affect *moaA* mRNA levels. The exact mechanism of translational activation therefore remains to be unraveled. Remarkably, the *moaA* mRNA region that contains one of the CsrA binding sites, can also form a molybdenum cofactor (MOCO) binding riboswitch. It is unclear whether MOCO and CsrA can bind simultaneously or whether MOCO prevents CsrA from binding (Patterson-Fortin et al., 2013). Lastly, CsrA induces premature transcription termination of the *pgaA* mRNA in *E. coli* by unfolding a secondary structure sequestering an entry site for transcription terminator factor Rho thereby regulating transcription elongation (Figueroa-Bossi et al., 2014). Although CsrA is predominantly involved in post-transcriptional regulation, the protein has recently been shown to affect transcription as well. In *P. protegens*, RsmA represses *lipA* transcription, although the mechanism remains unclear (Zha et al., 2014). Remarkably, *lipA* is additionally regulated by RsmE, which is one of the paralogs of RsmA in this species. RsmE blocks ribosome access by binding to the RBS of *lipA*.

Different *Pseudomonas* species indeed have three non-identical copies of RsmA, the CsrA ortholog in these bacteria. Although the expression profile of RsmE is slightly different from the one of RsmA, both proteins function in a largely redundant way (Reimmann et al., 2005). Another RsmA paralog is RsmN (also called RsmF). RsmN has a different structural organization of α-helices and β-sheets compared to RsmA and RsmE, but the tertiary structure is similar. This results in a conserved spatial organization of key residues within the dimeric structure, which is necessary for RNA-hairpin recognition (Marden et al., 2013; Morris et al., 2013). RsmA can bind to the mRNAs of its paralogs, thereby negatively influencing RsmE and RsmN protein expression (Reimmann et al., 2005; Marden et al., 2013). Altogether, these elements indicate that these proteins have a unique but overlapping regulatory role compared to RsmA. It has been suggested that variations in sequence, structure, RNA-binding affinities and specificities between these different paralogs facilitate tight gene-specific control at the global post-transcriptional level for Pseudomonads. The question remains why *E. coli* does not possess this wide array of CsrA paralogs (Morris et al., 2013).

### Hfq USES DIFFERENT MECHANISMS TO POST-TRANSCRIPTIONALLY REGULATE TARGET GENE EXPRESSION

Hfq is another very well-studied post-transcriptional regulator. The protein is widespread but not ubiquitously present throughout the bacterial kingdom (Sobrero and Valverde, 2012). In general, a knockout in *hfq* reduces the fitness of bacteria to survive in stressful environments (Tsui et al., 1994; Christiansen et al., 2004; Liu et al., 2010; Wang et al., 2014). Its role in RNA metabolism is more limited in Gram-positive bacteria compared to Gram-negative bacteria, which is illustrated by the fact that an *hfq* deletion does not have the same global effect on the transcriptome of *Bacillus*, like it has on the transcriptome of *E. coli* or *Salmonella* (Hämmerle et al., 2014).

Hfq has three different sites that can bind RNA: the distal, proximal and lateral site (Sauer, 2013). The binding preferences of these different RNA-binding sites are probably not that strict, as a study performed in *Listeria monocytogenes* shows that RNA-binding sites of Hfq have the potential to bind a wider variety of RNA sequences than was previously thought (Kovach et al., 2014). However, in *E. coli*, the distal site of Hfq does have a preference for repetitions of ARN-triplets or poly(A) stretches (Mikulecky et al., 2004; Link et al., 2009; Lorenz et al., 2010). The proximal site, on the other hand, has a preference for AU-rich single stranded sequences or poly(U) stretches (Schumacher et al., 2002; Moll et al., 2003). Finally, the lateral site of Hfq binds U-rich sequences and double stranded elements (Sauer et al., 2012), although the function of this lateral site in RNA-binding is controversial (Sauer, 2013). Some of the Hfq binding specificities overlap with the binding preferences of certain RNases. Therefore, Hfq can influence the RNase susceptibility of an mRNA or an sRNA. The AU-rich binding preference of the proximal binding site of Hfq, for example, is similar to the sequence that is recognized by RNaseE. Therefore, Hfq and RNaseE can compete for binding to the same region, reducing RNA decay (Massé et al., 2003; Moll et al., 2003; Mohanty et al., 2004). In the same way, Hfq plays a role in poly(A)-assisted RNA degradation. With its distal binding site, Hfq can bind to the poly(A) tail of mRNAs. Consequently, this region becomes inaccessible for exonucleases, like PNPase and RNaseII. Hfq binding at the poly(A) tail also impairs RNaseE processing. Both processes increase mRNA stability. However, binding of Hfq can also promote polyadenylation and thus promote poly(A)-assisted decay (Hajnsdorf and Régnier, 2000; Le Derout, 2003; Régnier and Hajnsdorf, 2013). Altogether, these mechanisms indicate how Hfq is important for the stability of sRNAs and mRNAs. Although Hfq is present at high levels in the cell, there is not enough Hfq to stabilize all sRNAs and mRNAs. Therefore, there is constant competition amongst sRNAs and mRNAs for Hfq binding (Vogel and Luisi, 2011).

Next to its role in regulating RNA stability, Hfq can function as a chaperone to stimulate the interaction of mRNAs and sRNAs (Møller et al., 2002; Zhang et al., 2002; Geissmann and Touati, 2004; Rasmussen et al., 2005; Kawamoto et al., 2006). Because there are different RNA-binding sites present in a hexameric Hfq molecule, it is possible that an sRNA and an mRNA are simultaneously bound to one Hfq molecule. Such binding to Hfq brings sRNAs and mRNAs in close proximity, enhancing the likelihood of interaction (Soper et al., 2011). However, cobinding of sRNAs and mRNAs to Hfq is transient and insufficient for sRNA-dependent regulation (Hopkins et al., 2011). Therefore RNA restructuring is also an important function of Hfq in this process (Henderson et al., 2013). The protein can change the secondary structure of the RNA molecules, making some regions in the mRNA more accessible for base pairing (Soper et al., 2011).

In addition to assisting sRNA/mRNA interactions, Hfq can also form a platform for the interaction of these RNA molecules with other proteins, e.g., RNaseE. This RNA/protein complex can then further interact with the other subunits of the RNaseE-based degradosome, causing degradation of the mRNA, and often also of the sRNA, in the complex (Morita et al., 2005; Aiba, 2007). The interaction between Hfq and RNaseE is most likely a combination of direct protein interactions between RNaseE and Hfq, which occur at the RhlB recognition region of RNaseE in the canonical degradosome (Ikeda et al., 2011), and indirect interactions via the RNA molecules they bind (De Lay et al., 2013). Hfq can indirectly interact in this way with a number of other proteins, like the cold shock protein CspC in *E. coli* and RsmA in *P. aeruginosa*, a protein that was discussed previously in this review (Sorger-Domenigg et al., 2007; Cohen-Or et al., 2010). Another protein that may be recruited by Hfq is Crc, a protein involved in catabolite repression control in *P. fluorescens* and *P. aeruginosa*. Crc was originally identified as an RBP, able to bind short unpaired A-rich motifs (AAnAAnAA) at or near the RBS, thereby inhibiting translation initiation (Moreno et al., 2009; Sonnleitner et al., 2009; Browne et al., 2010). However, a recent study shows that Crc has no RNA-binding capacity and previous results on RNA-binding rely on contaminations of Crc protein samples with Hfq (Milojevic et al., 2013). Hfq and Crc are now assumed to cooperate for binding to RNAs that contain an A-rich motif, because both proteins form a cocomplex and are both necessary for catabolite repression. Possibly Hfq recruits Crc or Crc modifies Hfq in such a way that it can more efficiently bind to the A-rich stretches with its distal site, preventing translation (Moreno et al., 2014).

Next to its functions as a (de)stabilizing factor for RNA molecules and as an interaction platform for RNA/RNA an RNA/protein interactions, Hfq carries out other functions. One of these functions is competing with initiating ribosomes for access to the RBS by binding AU-rich regions close to the RBS which are acting as translational enhancers (Vytvytska et al., 2000; Desnoyers and Masse, 2012). These translational enhancers facilitate the interaction of an mRNA with protein S1 near the RBS, but this interaction is impaired when Hfq is bound. S1 is a protein, weakly associated to the 30S subunit of the ribosome that facilitates the recognition of mRNAs by ribosomes at the initial step of translation (Subramanian, 1983). Sometimes, sRNAs are involved in this regulatory process. Spot42, for example, recruits Hfq at the enhancer region (Desnoyers and Masse, 2012). In another case, Hfq binding takes place without an sRNA as a recruiting molecule. Oppositely, binding of the sRNA RyhB can prevent Hfq from binding to the enhancer region (Salvail et al., 2013). In addition, Hfq has a role in transcription regulation, by inhibiting the function of the Rho protein, which is involved in transcription termination (Le Derout et al., 2010; Rabhi et al., 2011).

While CsrA and Hfq use different mechanisms to globally regulate gene expression, there are proteins that have only been shown to use a confined number of these mechanisms or regulate a more limited number of genes. They will be described hereafter.

### RNA-BINDING PROTEINS ADAPTING THE SUSCEPTIBILITY TO RNases

In addition to its role in facilitating ribosome interactions with the mRNA, protein S1 also has a function in post-transcriptional regulation. The protein can stabilize RNA molecules by directly shielding RNase recognition sites (Hajnsdorf and Boni, 2012). Although S1 has no strict sequence specificity, it does have a higher affinity for AU-rich mRNA sites. As RNaseE preferentially binds AU-rich single stranded regions as well, S1 can shield RNaseE recognition sites and protect mRNAs against cleavage (Komarova et al., 2005). Because S1 is capable of binding to sRNAs with the same affinity as Hfq, it has been suggested that S1 can theoretically regulate sRNA stability as well. However, the biological relevance of this suggestion still needs to be elucidated (Koleva et al., 2006; Windbichler et al., 2008).

S1 directly competes with RNases for binding to specific sequences. However, other proteins affect mRNA stability by promoting a change in the secondary structure of their mRNA targets. Consequently, RNase recognition sites become more or less available for RNases. An example of proteins that use this mechanism are cold shock proteins. These proteins are induced when bacteria encounter a temperature downshift. One of the physiological effects of cold is the stabilization of secondary structures that make RNase recognition sites inaccessible, which likewise impairs RNA degradation. Binding of the cold shock protein, CspA, to these mRNAs causes, together with the induced helicases, cold-induced secondary structures in the mRNA to be melted. Adversely, cold shock proteins can also prevent RNA degradation. CspE can bind poly(A) sites and can consequently interfere with either binding of PNPase or with internal cleavage by RNaseE (Feng et al., 2001). Additionally, cold shock proteins can assist in unwinding of secondary structures that sequester the RBS, which enhances translation efficiency (Barria et al., 2013).

### RNA-BINDING PROTEINS THAT AFFECT sRNA STABILITY

Hfq is very well known for its role in regulating sRNA and mRNA stability. However, recently, other RBPs were identified that regulate the stability of specific sRNAs. A first example is CsrD (Suzuki et al., 2006). In *E. coli*, this protein is involved in the turnover of the sRNAs CsrB and CsrC. These sRNAs regulate the activity of CsrA, which is an RBP that was described earlier. Although CsrD destabilizes the sRNAs CsrB and CsrC in an RNaseE-dependent way, these sRNAs have no obvious RNaseE recognition sites and CsrD has no RNase activity itself. Therefore, it was suggested that CsrD might induce structural changes in the sRNA, making it more susceptible for RNaseE (Suzuki et al., 2006). CsrD does not bind specifically to CsrB or CsrC, however, the action of CsrD seems to be specific. This indicates that there are additional factors in the cell that determine the specificity of the process (Suzuki et al., 2006). A second example of an RBP specifically regulating sRNA stability is RapZ. RapZ is a protein identified in *E. coli* that functions as an adaptor protein guiding the processing of the sRNA, GlmZ. The protein has been reported to recruit RNaseE to the sRNA. It has been hypothesized that this occurs through changing the structure of the sRNA so it can be recognized by RNaseE, or by functioning as an interaction platform by delivering the sRNA to RNaseE (Göpel et al., 2013). Most likely, there are more proteins that bind sRNAs and target them for degradation, just like RapZ and CsrD.

### RNA-BINDING PROTEINS THAT MODULATE RBS ACCESSIBILITY

TRAP (trp RNA-binding attenuation protein), a protein involved in the regulation of tryptophan metabolism of *B. subtilis*, acts through modulating RBS accessibility for ribosomes. The protein specifically binds multiple (9–11) NAG repeats, separated by non-conserved spacers. Because of the extended recognition sequence, TRAP can regulate a small subset of genes, all involved in tryptophan metabolism. TRAP can act as a post-transcriptional regulator by directly blocking ribosome access to the RBS (Babitzke et al., 1994; Yang et al., 1995; Du et al., 1997; Sarsero et al., 2000; Yakhnin et al., 2004). Although the protein binds multiple NAG repeats, it is sufficient that one repeat overlaps with the RBS to block ribosome access to this region (Babitzke et al., 1994; Babitzke et al., 1995). TRAP can also promote a change in secondary structure which influences the availability of the RBS for a subset of genes (Merino et al., 1995; Du and Babitzke, 1998). Additionally, the protein is involved in the regulation of transcription elongation, which will be described later.

The regulatory RBP BpuR from *Borrelia burgdorferi* possibly acts by competing with ribosome binding as well, as the protein has been shown to bind to the 5′ region of its mRNA target, thereby blocking translation. However, the only identified RNA target of this protein this far is its own mRNA. Most likely, BpuR is a post-transcriptional regulator of other genes in the *Borrelia* genome (Jutras et al., 2013b). BpuR can also act as a DNA binding protein, but it binds RNA with higher affinity (Jutras et al., 2013a).

### PROTEINS ACTING AS AN INTERACTION PLATFORM WITH OTHER MOLECULES

YbeY is a widely conserved protein, known to influence the maturation of rRNAs and to be involved in the quality control of 70S ribosomes (Davies et al., 2011; Jacob et al., 2014). Although it is an essential RNase in some bacteria like *Vibrio cholera*, the protein is not essential and has weak RNase activity in *E. coli* and *Sinorhizobium meliloti* (Vercruysse et al., 2014). In these bacteria, both *in silico* and phenotypic indications exist, supporting the hypothesis that YbeY plays a role in sRNA regulation, although YbeY has not yet been shown to actually bind sRNAs *in vivo* in these species. *In silico* analyses show that YbeY displays high sequence and structural similarities to MID domains of Argonaut proteins, the central component of sRNA-mediated gene silencing in eukaryotes. Argonaut proteins bind sRNAs that function as sequence-specific guides to lead the Argonaut proteins to perfectly or partially complementary sequences (Mallory and Vaucheret, 2010). Additionally, structural models assigned a probable RNA-binding site for YbeY. Phenotypically, there are striking similarities between an *smc01113* mutant, the *ybeY* ortholog in *S. meliloti*, and an *hfq* mutant (Pandey et al., 2011). Mutated YbeY indeed causes an increased sensitivity to various stresses, similarly to when Hfq is mutated. Moreover, YbeY modulates the levels of both already identified Hfq-dependent and Hfq-independent sRNAs and their targets in *E. coli* (Pandey et al., 2014), which suggests that YbeY has a central role in RNA metabolism. The exact working mechanism of the YbeY protein remains unknown. Besides the suggested role in the interaction of mRNAs and sRNAs, YbeY might still have a catalytic role as an RNase as a functional equivalent of RNaseE (Vercruysse et al., 2014).

While YbeY may play a central role in the general sRNA metabolism, there are some RBPs that form a platform of interaction for a more limited number of sRNAs. One example is the FinO family of bacterial chaperones. FinO is involved in the regulation of gene expression from the F-plasmid by facilitating the interaction between the sRNA FinP and the mRNA of the F plasmid transcription factor *traJ*. The protein facilitates sRNA-mRNA interactions by destabilizing internal hairpins in target RNAs (Arthur et al., 2003) and protects them from RNaseE (Jerome et al., 1999). However, contrary to Hfq, FinO does not simultaneously bind mRNA and sRNA molecules (Chaulk et al., 2010). Other proteins assigned to this FinO-family include NMB1681 in *Neisseria meningitides* and ProQ in *E. coli*. Although NMB1681 has been shown to restore phenotypes in an *E. coli finO* mutant, the role of this protein in *Neisseria* has not been unraveled (Chaulk et al., 2010). ProQ has an N-terminus homologous to FinO and a C-terminus homologous to the C-terminus of Hfq. Both the N- and the C-terminus facilitate sRNA-mRNA interactions. However, the Hfq-like domain most likely assists in sRNA–mRNA interaction, while the FinO-like domain confers sequence-specific properties to the protein (Chaulk et al., 2011; Sheidy and Zielke, 2013). ProQ also associates with the ribosome, which appears to be mediated by an interaction between ProQ and its targets being translated (Sheidy and Zielke, 2013).

A second example of an RBP possibly involved in assisting a limited number of sRNA–mRNA interactions is FbpB. FbpB is a small protein involved in the regulation of iron metabolism in *B. subtilis*. The protein is suggested to function as a coregulator of the translational repressor FsrA, by targeting the sRNA FsrA to specific transcripts and increasing the effectiveness of the sRNA. The phenotype of an *fbpB* mutant can indeed be restored by an upregulation of FsrA. FbpB is possibly involved in the recruitment of the degradation machinery of *B. subtilis* as well, thereby causing degradation of the sRNA–mRNA complex (Smaldone et al., 2012). Although this is still speculative, this would be consistent with the functions of Hfq in *E. coli*.

### RNA-BINDING PROTEINS THAT MODULATE TRANSCRIPTION TERMINATOR/ANTITERMINATOR FORMATION

TRAP and cold shock proteins have been described earlier as they can induce a change in the secondary structure of their RNA target which modulates RNA stability or translation initiation efficiency. Additionally, these proteins can modulate transcription elongation by stabilizing a transcription terminator or antiterminator structure upon binding. TRAP, activated by tryptophan, binds to the *Bacillus trp* 5′ leader transcript and occludes the formation of the antiterminator. This antiterminator is located upstream of a terminator and the formation of both structures is mutually exclusive. Thereby, TRAP binding enables the terminator to be formed and transcription is prematurely stopped (Babitzke, 2004). The cold shock proteins CspA and homologs CspC and CspE from *E. coli*, work in the opposite way. These proteins prevent the formation of transcription terminators by stabilizing an antiterminator structure (Bae et al., 2000; Phadtare et al., 2002).

Another example of RBPs that stabilize an antiterminator structure upon binding is the Bgl/Sac family. These proteins are widely distributed and recognize a 23–30 nucleotide stretch called ribonucleotide antiterminator (RAT) that partially overlaps with the terminator sequence (Aymerich and Steinmetz, 1992). Similarly to cold shock proteins, binding of an RBP of the Bgl/Sac family causes this antiterminator region to fold in a stem-loop structure that occludes the formation of the terminator. The Bgl sytem of *E. coli* was the first mechanism that involves protein-mediated antitermination (Mahadevan and Wright, 1987; Schnetz and Rak, 1988). Members of this family have been identified in different bacteria such as *E. coli*, *B. subtilis*, *Lactococcus lactis*, and *Erwinia chrysanthemi.* They control the expression of genes required for the utilization of carbohydrates (Rutberg, 1997).

Lastly, PyrR of *B. subtilis* is involved in modulating transcription terminator/antiterminator formation, although its mechanism of action is a little different compared to the examples described above. PyrR, activated in the presence of uridine, stabilizes an anti-antiterminator structure. This structure sequesters nucleotides of the antiterminator by basepairing with sequences that lie further upstream, inducing terminator formation and preventing gene expression. Only in the absence of uridine the antiterminator can form and expression of the RNA target is possible (Lu et al., 1996).

### POST-TRANSCRIPTIONALLY ACTIVE REGULATORY PROTEINS WITH AN UNKNOWN MECHANISM

Other post-transcriptionally active regulatory proteins have been identified. However, often their exact mechanism of action remains unclear. For example, YopD is a component of the type III secretion system (T3SS) of *Yersinia* species and is conserved in pathogens with a T3SS (Schiano and Lathem, 2012). It translocates virulence factors across the cell membrane of the host. Simultaneously, YopD post-transcriptionally regulates genes of the T3SS directly and specifically in complex with LcrH, a secretion chaperone (Schiano and Lathem, 2012). The specificity of YopD is thought to be based on the interaction of the protein with short AU-rich sequences, both up- and downstream of the start codon of the target genes. However, the interaction is more complex than YopD/LcrH and AU sequences alone. Other interaction partners are involved. The post-transcriptionally active complex of YopD, LcrH and other interacting components binds to the 5′UTR of its targets and represses their translation, but the mechanism remains unclear. YopD has been suggested to facilitate degradation of these targets by directly competing with ribosome binding or promoting degradation (Chen and Anderson, 2011). However, a recent report shows that YopD affects translation by modifying the ribosome itself (Kopaskie et al., 2013). It is remarkable that a protein with a structural function has a regulatory role as well.

In *Shigella sonnei*, RodZ was identified as a membrane-localized cytoskeletal protein that retains the rod-shaped morphology of the bacterium. Later, this protein was also shown to be involved in post-transcriptional regulation, as the protein has RNA-binding capacity and its expression leads to repression of InvE protein synthesis by means of a decreased stability of the mRNA. However, the working mechanism still needs to be resolved. Possibly it forms a platform where mRNAs and other putative regulatory factors coincide (Mitobe et al., 2011).

FlbT has been identified in *Caulobacter crescentus* and *Brucella melitensis*, where it is proposed to bind to the 5′UTR of the *fliC* mRNA. However, it is not clear how it post-transcriptionally regulates gene expression. In *Caulobacter* binding of FlbT promotes degradation of its mRNA targets, whereas in *Brucella* FlbT is proposed to be an activator of gene expression (Anderson and Gober, 2000; Ferooz et al., 2011).

The family of AmiR and NasR transcriptional antiterminator regulator (ANTAR) domain proteins is a last example of RBPs acting at the post-transcriptional level with an undefined regulatory mechanism. They are widely distributed among different species and are involved in transcription antitermination. These proteins recognize an RNA motif consisting of two tandem stem-loops. However the exact molecular mechanism of antitermination has not been determined (Ramesh et al., 2012). For NasR of *Klebsiella oxytoca*, it has been suggested that it does not involve the formation of an antitermination structure (Chai and Stewart, 1999).

## POST-TRANSCRIPTIONAL REGULATION OF POST-TRANSCRIPTIONALLY ACTIVE REGULATORY PROTEINS

To ensure that gene expression is adjusted according to the needs of the cell, it is crucial that the expression and the activity of the regulatory RBPs themselves are tightly controlled as well. Remarkably, protein expression or protein activity is regulated itself by post-transcriptional regulatory processes. Proteins that modulate RBS accessibility, like CsrA and BpuR, have their recognition site present in the 5′UTR of their own mRNA. Protein binding to the mRNA consequently competes with ribosome binding, reducing translation initiation efficiency and the expression of the protein (Yakhnin et al., 2011b; Jutras et al., 2013b). Similarly, Hfq expression is autoregulated. The protein inhibits ribosome binding to its own mRNA, making it at the same time more vulnerable for cleavage by RNaseE. Two Hfq binding sites were identified upstream of the start codon, with one overlapping with the RBS. However, the other binding site is also necessary for translational repression together with a hairpin structure in the coding region. These elements possibly function as stabilizing elements for RNA/protein interaction (Vecerek et al., 2005).

Secondly, the activity of different RBPs is regulated with sRNAs. These sRNAs carry multiple high affinity sequences that are specifically recognized by a regulatory RBP. Binding of the sRNA sequesters the protein, resulting in a lower number of proteins available for binding to its mRNA targets. The best studied examples of sRNAs that regulate protein activity by mimicking the protein binding sequence are the sRNAs CsrB and CsrC (Liu et al., 1997; Weilbacher et al., 2003). They bind to the global regulator CsrA, which was discussed previously. CsrB and CsrC carry 18 and 9 CsrA binding sequences, respectively. Recently, another *E. coli* sRNA, McaS, was shown to bind CsrA. This sRNA has at least two CsrA binding sites (Jørgensen et al., 2013). The different sRNA molecules that regulate CsrA activity are differentially expressed in some conditions. Consequently, the activity of the RBP can be regulated in response to different environmental conditions (Jørgensen et al., 2013). Remarkably, CsrA activity is not only regulated by sRNAs. In *Salmonella* Typhimurium, for example, the *fim* mRNA can inhibit CsrA function as well. This mRNA carries the CsrA recognition sequence but the stability or the translation of *fim* mRNA is not affected by CsrA binding, excluding it from being a regulated CsrA target (Sterzenbach et al., 2013).

While CsrA is the best studied example of a protein that is regulated by sRNAs, more of them are known. The Hfq protein, which was extensively discussed above, is regulated by the sRNA CrcZ in *E. coli*. The sRNA has multiple A-rich stretches to which Hfq can potentially bind with its distal RNA-binding region. As Hfq has other RNA-binding sites it is possible that the protein can still bind and regulate other RNA molecules when CrcZ is bound to this distal site. However, this remains to be resolved (Sonnleitner and Bläsi, 2014). A last example is RapZ, which is sequestered by binding to the sRNA GlmY. This sRNA resembles the mRNA target of RapZ, i.e., GlmZ, by a conserved central stem-loop structure (Göpel et al., 2013). This shows that not only the global regulators, like CsrA and Hfq, are regulated through sRNA mimicry.

Although the activity of some RBPs is regulated by sRNAs, the activity of others is still regulated by proteins. This is the case for the TRAP protein, which is regulated by an anti-TRAP protein that binds near the RNA-binding pocket of the TRAP protein, preventing TRAP from binding to its mRNA targets (Snyder et al., 2004). CsrA from *B. subtilis* is another RBP that is regulated by a protein, FliW. This antagonistic protein binds near the active site of the protein. Remarkably, a CsrB-like sRNA has also been identified in *B. subtilis* (Mukherjee et al., 2011). Its regulatory role and the importance compared to FliW has not been unraveled yet (Kulkarni et al., 2006).

## CONCLUSION AND PERSPECTIVES

Regulation of gene expression is very complex and takes place at different regulatory levels. At the post-transcriptional level, RBPs are important gene expression regulators. These proteins generally act by adapting RNA stability and mRNA translation efficiency. Hereto, they use different mechanisms, including (i) adaptation of the susceptibility of the target RNAs for RNases, (ii) modulation of the accessibility of the RBS for ribosome binding, (iii) acting as a chaperone for the interaction of the RNA target with other effector molecules, and (iv) modulation of transcription terminator/antiterminator formation. Different post-transcriptionally active RBPs have been identified in *E. coli* and in other bacteria, with Hfq and CsrA being the two best studied examples. These proteins apply different mechanisms to regulate the expression of their target genes. Recently, additional RBPs that affect mRNA and/or sRNA stability or translation efficiency have been identified. Although many mechanistic questions remain, they use mostly similar regulatory mechanisms to regulate the expression of their targets. Additionally, they are post-transcriptionally regulated themselves through autoregulation and regulation by sRNA mimicry.

A number of post-transcriptionally active RBPs have been identified. However, it is very likely that more RBPs are active as post-transcriptional regulators, given that some known regulatory RBPs have another function, e.g., as a transcription regulator or a structural protein. Moreover, proteins that specifically regulate sRNA stability have only recently been discovered. Therefore, most likely, more proteins of this class will be identified in the future. Additionally, relatively little is known about this type of regulatory proteins in other bacteria than *E. coli*. Therefore, methods have been developed to identify new RBPs. One of these methods is the *in vitro* or *in vivo* assembly of RNA and RBPs, followed by mass spectrometry (Tsai et al., 2011). This method has already been optimized for *Helicobacter pylori*, *E. coli*, *Salmonella* Typhimurium and *P. aeruginosa* (Windbichler et al., 2008; Said et al., 2009; Rieder et al., 2012; Osborne et al., 2014).

Further studies on well-known and newly identified post-transcriptionally active proteins will lead to a better understanding of how bacteria use this type of gene regulation to respond to changes in the environment and how different post-transcriptional networks interact with transcriptional regulons and with each other. This knowledge will create opportunities for new or improved biotechnological applications, e.g., in synthetic biology as a tool to control gene expression, complementing the current approaches of transcription control. Additionally, as many of these proteins play a central role in RNA metabolism, interfering with the expression or the function of these proteins can be interesting as an alternative antimicrobial strategy.

## Conflict of Interest Statement

The authors declare that the research was conducted in the absence of any commercial or financial relationships that could be construed as a potential conflict of interest.

## References

[B1] Aiba H. (2007). Mechanism of RNA silencing by Hfq-binding small RNAs. *Curr. Opin. Microbiol.* 10 134–139 10.1016/j.mib.2007.03.010 17383928

[B2] Anderson P. E. Gober J. W. (2000). FlbT, the post-transcriptional regulator of flagellin synthesis in Caulobacter crescentus, interacts with the 5’ untranslated region of flagellin mRNA. *Mol. Microbiol.* 38 41–52 10.1046/j.1365-2958.2000.02108.x 11029689

[B3] Arthur D. C. Ghetu A. F. Gubbins M. J. Edwards R. A. Frost L. S. Glover J. N. M. (2003). FinO is an RNA chaperone that facilitates sense-antisense RNA interactions. *EMBO J.* 22 6346–6355 10.1093/emboj/cdg607 14633993 PMC291848

[B4] Aymerich S. Steinmetz M. (1992). Specificity determinants and structural features in the RNA target of the bacterial antiterminator proteins of the BglG / SacY family. *Proc. Natl. Acad. Sci. U.S.A.* 89 10410–10414 10.1073/pnas.89.21.10410 1279678 PMC50348

[B5] Babitzke P. (2004). Regulation of transcription attenuation and translation initiation by allosteric control of an RNA-binding protein: the *Bacillus subtilis* TRAP protein. *Curr. Opin. Microbiol.* 7 132–139 10.1016/j.mib.2004.02.003 15063849

[B6] Babitzke P. Bear D. G. Yanofsky C. (1995). TRAP, the trp RNA-binding attenuation protein of *Bacillus subtilis*, is a toroid-shaped molecule that binds transcripts containing GAG or UAG repeats separated by two nucleotides. *Proc. Natl. Acad. Sci. U.S.A.* 92 7916–7920 10.1073/pnas.92.17.7916 7544009 PMC41257

[B7] Babitzke P. Stults J. T. Shire S. J. Yanofsky C. (1994). TRAP, the trp RNA-binding attenuation protein of *Bacillus subtilis*, is a multisubunit complex that appears to recognize G/UAG repeats in the trpEDCFBA and trpG transcripts. *J. Biol. Chem.* 269 16597–16604. 7515880

[B8] Bachem S. Stülke J. (1998). Regulation of the *Bacillus subtilis* GlcT antiterminator protein by components of the phosphotransferase system. *J. Bacteriol.* 180 5319–5326. 9765562 10.1128/jb.180.20.5319-5326.1998 PMC107579

[B9] Bae W. Xia B. Inouye M. Severinov K. (2000). *Escherichia coli* CspA-family RNA chaperones are transcription antiterminators. *Proc. Natl. Acad. Sci. U.S.A.* 97 7784–7789 10.1073/pnas.97.14.7784 10884409 PMC16622

[B10] Baker C. S. Eöry L. A. Yakhnin H. Mercante J. Romeo T. Babitzke P. (2007). CsrA inhibits translation initiation of *Escherichia coli* hfq by binding to a single site overlapping the Shine-Dalgarno sequence. *J. Bacteriol.* 189 5472–5481 10.1128/JB.00529 17526692 PMC1951803

[B11] Baker C. S. Morozov I. Suzuki K. Romeo T. Babitzke P. (2002). CsrA regulates glycogen biosynthesis by preventing translation of glgC in *Escherichia coli*. *Mol. Microbiol.* 44 1599–1610 10.1046/j.1365-2958.2002.02982.x 12067347

[B12] Barria C. Malecki M. Arraiano C. M. (2013). Bacterial adaptation to cold. *Microbiology* 159 2437–2443 10.1099/mic.0.052209-0 24068238

[B13] Beyer D. Skripkin E. Wadzack J. Nierhaus K. H. (1994). How the ribosome moves along the mRNA during protein synthesis. *J. Biol. Chem.* 269 30713–30717. 7982992

[B14] Brencic A. Lory S. (2009). Determination of the regulon and identification of novel mRNA targets of *Pseudomonas aeruginosa* RsmA. *Mol. Microbiol.* 72 612–632 10.1111/j.1365-2958.2009.06670.x 19426209 PMC5567987

[B15] Browne P. Barret M. O’Gara F. Morrissey J. P. (2010). Computational prediction of the Crc regulon identifies genus-wide and species-specific targets of catabolite repression control in *Pseudomonas* bacteria. *BMC Microbiol.* 10:300 10.1186/1471-2180-10-300 PMC3003667 21108798

[B16] Burrowes E. Baysse C. Adams C. O’Gara F. (2006). Influence of the regulatory protein RsmA on cellular functions in *Pseudomonas aeruginosa* PAO1, as revealed by transcriptome analysis. *Microbiology* 152 405–418 10.1099/mic.0.28324-0 16436429

[B17] Callaghan A. J. Marcaida M. J. Stead J. A. McDowall K. J. Scott W. G. Luisi B. F. (2005). Structure of *Escherichia coli* RNase E catalytic domain and implications for RNA turnover. *Nature* 437 1187–1191 10.1038/nature04084 16237448

[B18] Carpousis A. J. (2007). The RNA degradosome of *Escherichia coli*: an mRNA-degrading machine assembled on RNase E. *Annu. Rev. Microbiol.* 61 71–87 10.1146/annurev.micro.61.080706.093440 17447862

[B19] Chai W. Stewart V. (1999). RNA sequence requirements for NasR-mediated, nitrate-responsive transcription antitermination of the *Klebsiella oxytoca* M5al nasF operon leader. *J. Mol. Biol.* 292 203–216 10.1006/jmbi.1999.3084 10493869

[B20] Chaulk S. Lu J. Tan K. Arthur D. C. Edwards R. A. Frost L. S. (2010). *N. meningitidis* 1681 is a member of the FinO family of RNA chaperones. *RNA Biol.* 7 812–819 10.4161/rna.7.6.13688 21045552 PMC3073339

[B21] Chaulk S. G. Smith-Frieday M. N. Arthur D. C. Culham D. E. Edwards R. A. Soo P. (2011). ProQ is an RNA chaperone that controls ProP levels in *Escherichia coli*. *Biochemistry* 50 3095–3106 10.1021/bi101683a 21381725

[B22] Chen Y. Anderson D. M. (2011). Expression hierarchy in the *Yersinia* type III secretion system established through YopD recognition of RNA. *Mol. Microbiol.* 80 966–980 10.1111/j.1365-2958.2011.07623.x 21481017 PMC4128491

[B23] Christiansen J. K. Larsen M. H. Ingmer H. Søgaard-andersen L. Kallipolitis B. H. (2004). The RNA-binding protein Hfq of *Listeria monocytogenes*: role in stress tolerance and virulence. *J. Bacteriol.* 186 3355–3362 10.1128/JB.186.11.3355-3362.2004 15150220 PMC415768

[B24] Cohen-Or I. Shenhar Y. Biran D. Ron E. Z. (2010). CspC regulates rpoS transcript levels and complements hfq deletions. *Res. Microbiol.* 161 694–700 10.1016/j.resmic.2010.06.009 20633642

[B25] Davies B. W. Köhrer C. Jacob A. I. Simmons L. A. Zhu J. Aleman L. M. (2011). Role of *Escherichia coli* YbeY, a highly conserved protein, in rRNA processing. *Mol. Microbiol.* 78 506–518 10.1111/j.1365-2958.2010.07351.x 20807199 PMC2959132

[B26] Deana A. Belasco J. G. (2005). Lost in translation: the influence of ribosomes on bacterial mRNA decay. *Genes Dev.* 19 2526–2533 10.1101/gad.1348805 16264189

[B27] De Lay N. Schu D. J. Gottesman S. (2013). Bacterial small RNA-based negative regulation: Hfq and its accomplices. *J. Biol. Chem.* 288 7996–8003 10.1074/jbc.R112.441386 23362267 PMC3605619

[B28] Desnoyers G. Bouchard M. P. Massé E. (2013). New insights into small RNA-dependent translational regulation in prokaryotes. *Trends Genet.* 29 92–98 10.1016/j.tig.2012.10.004 23141721

[B29] Desnoyers G. Masse E. (2012). Noncanonical repression of translation initiation through small RNA recruitment of the RNA chaperone Hfq. 42 726–739 10.1101/gad.182493.111 PMC3323883 22474262

[B30] Du H. Babitzke P. (1998). trp RNA-binding attenuation protein-mediated long distance RNA refolding regulates translation of trpE in *Bacillus subtilis*. *J. Biol. Chem.* 273 20494–20503 10.1074/jbc.273.32.20494 9685405

[B31] Du H. Tarpey R. Babitzke P. (1997). The trp RNA-binding attenuation protein regulates TrpG synthesis by binding to the trpG ribosome binding site of *Bacillus subtilis*. *J. Bacteriol.* 179 2582–2586. 9098056 10.1128/jb.179.8.2582-2586.1997 PMC179007

[B32] Dubey A. K. Baker C. S. Romeo T. Babitzke P. (2005). RNA sequence and secondary structure participate in high-affinity CsrA – RNA interaction. *RNA* 11 1579–1587 10.1261/rna.2990205.3 16131593 PMC1370842

[B33] Dubey A. K. Baker C. S. Suzuki K. Jones A. D. Pandit P. Romeo T. (2003). CsrA regulates translation of the *Escherichia coli* carbon starvation gene, cstA, by blocking ribosome access to the cstA transcript. *J. Bacteriol.* 185 4450–4460 10.1128/JB.185.15.4450-4460.2003 12867454 PMC165747

[B34] Edwards A. N. Patterson-Fortin L. M. Vakulskas C. A. Mercante J. W. Potrykus K. Vinella D. (2011). Circuitry linking the Csr and stringent response global regulatory systems. *Mol. Microbiol.* 80 1561–1580 10.1111/j.1365-2958.2011.07663.x 21488981 PMC3115499

[B35] Even S. Pellegrini O. Zig L. Labas V. Vinh J. Bréchemmier-Baey D. (2005). Ribonucleases J1 and J2: two novel endoribonucleases in *B. subtilis* with functional homology to *E. coli* RNase E. *Nucleic Acids Res.* 33 2141–2152 10.1093/nar/gki505 15831787 PMC1079966

[B36] Feng Y. Huang H. Liao J. Cohen S. N. (2001). *Escherichia coli* poly(A)-binding proteins that interact with components of degradosomes or impede RNA decay mediated by polynucleotide phosphorylase and RNase E. *J. Biol. Chem.* 276 31651–31656 10.1074/jbc.M102855200 11390393

[B37] Ferooz J. Lemaire J. Letesson J.-J. (2011). Role of FlbT in flagellin production in *Brucella melitensis*. *Microbiology* 157 1253–1262 10.1099/mic.0.044867-0 21273249

[B38] Figueroa-Bossi N. Schwartz A. Guillemardet B. D’Heygère F. Bossi L. Boudvillain M. (2014). RNA remodeling by bacterial global regulator CsrA promotes Rho-dependent transcription termination. *Genes Dev.* 28 1239–1251 10.1101/gad.240192.114 24888591 PMC4052769

[B39] Finn R. D. Bateman A. Clements J. Coggill P. Eberhardt R. Y. Eddy S. R. (2014). Pfam: the protein families database. *Nucleic Acids Res.* 42 D222–D2230 10.1093/nar/gkt1223 24288371 PMC3965110

[B40] Folichon M. (2003). The poly(A) binding protein Hfq protects RNA from RNase E and exoribonucleolytic degradation. *Nucleic Acids Res.* 31 7302–7310 10.1093/nar/gkg915 14654705 PMC291859

[B41] Gaballa A. Antelmann H. Aguilar C. Khakh S. K. Song K.-B. Smaldone G. T. (2008). The *Bacillus subtilis* iron-sparing response is mediated by a Fur-regulated small RNA and three small, basic proteins. *Proc. Natl. Acad. Sci. U.S.A.* 105 11927–11932 10.1073/pnas.0711752105 18697947 PMC2575260

[B42] Geissmann T. A. Touati D. (2004). Hfq, a new chaperoning role: binding to messenger RNA determines access for small RNA regulator. *EMBO J.* 23 396–405 10.1038/sj.emboj.7600058 14739933 PMC1271764

[B43] Gollnick P. Babitzke P. Antson A. Yanofsky C. (2005). Complexity in regulation of tryptophan biosynthesis in *Bacillus subtilis*. *Annu. Rev. Genet.* 39 47–68 10.1146/annurev.genet.39.073003.093745 16285852

[B44] Göpel Y. Papenfort K. Reichenbach B. Vogel J. Görke B. (2013). Targeted decay of a regulatory small RNA by an adaptor protein for RNase E and counteraction by an anti-adaptor RNA. *Genes Dev.* 27 552–564 10.1101/gad.210112.112 23475961 PMC3605468

[B45] Hajnsdorf E. Boni I. V. (2012). Multiple activities of RNA-binding proteins S1 and Hfq. *Biochimie* 94 1544–1553 10.1016/j.biochi.2012.02.010 22370051

[B46] Hajnsdorf E. Régnier P. (2000). Host factor Hfq of *Escherichia coli* stimulates elongation of poly(A) tails by poly(A) polymerase I. *Proc. Natl. Acad. Sci. U.S.A.* 97 1501–1505 10.1073/pnas.040549897 10677490 PMC26463

[B47] Hämmerle H. Amman F. Veèerek B. Stülke J. Hofacker I. Bläsi U. (2014). Impact of Hfq on the *Bacillus subtilis* transcriptome. *PLoS ONE* 9:e98661 10.1371/journal.pone.0098661 PMC4059632 24932523

[B48] Henderson C. A. Vincent H. A. Casamento A. Stone C. M. Phillips J. O. Cary P. D. (2013). Hfq binding changes the structure of *Escherichia coli* small noncoding RNAs OxyS and RprA, which are involved in the riboregulation of rpoS. *RNA* 19 1089–1104 10.1261/rna.034595.112 23804244 PMC3708529

[B49] Herschlag D. (1995). RNA chaperones and the RNA folding problem. *J. Biol. Chem.* 270 20871–20874 10.1074/jbc.270.36.20871 7545662

[B50] Hopkins J. F. Panja S. Woodson S. A. (2011). Rapid binding and release of Hfq from ternary complexes during RNA annealing. *Nucleic Acids Res.* 39 5193–5202 10.1093/nar/gkr062 21378124 PMC3130257

[B51] Huttenhofer A. Fnolier H. (1994). Footprinting mRNA- ribosome complexes with chemical probes. *EMBO J.* 13 3892–3901. 8070416 10.1002/j.1460-2075.1994.tb06700.x PMC395302

[B52] Ikeda Y. Yagi M. Morita T. Aiba H. (2011). Hfq binding at RhlB-recognition region of RNase E is crucial for the rapid degradation of target mRNAs mediated by sRNAs in *Escherichia coli*. *Mol. Microbiol.* 79 419–432 10.1111/j.1365-2958.2010.07454.x 21219461

[B53] Irie Y. Starkey M. Edwards A. N. Wozniak D. J. Romeo T. Parsek M. R. (2010). *Pseudomonas aeruginosa* biofilm matrix polysaccharide Psl is regulated transcriptionally by RpoS and post-transcriptionally by RsmA. *Mol. Microbiol.* 78 158–172 10.1111/j.1365-2958.2010.07320.x 20735777 PMC2984543

[B54] Jacob A. I. Köhrer C. Davies B. W. Rajbhandary U. L. Walker G. C. (2014). Conserved bacterial RNase YbeY plays key roles in 70S ribosome quality control and 16S rRNA maturation. *Mol. Cell* 49 427–438 10.1016/j.molcel.2012.11.025 23273979 PMC3570609

[B55] Jerome L. J. van Biesen T. Frost L. S. (1999). Degradation of FinP antisense RNA from F-like plasmids: the RNA-binding protein, FinO, protects FinP from ribonuclease E. *J. Mol. Biol.* 285 1457–1473 10.1006/jmbi.1998.2404 9917389

[B56] Jonas K. Edwards A. N. Simm R. Romeo T. Römling U. Melefors O. (2008). The RNA binding protein CsrA controls cyclic di-GMP metabolism by directly regulating the expression of GGDEF proteins. *Mol. Microbiol.* 70 236–257 10.1111/j.1365-2958.2008.06411.x 18713317 PMC2735045

[B57] Jørgensen M. G. Thomason M. K. Havelund J. Valentin-Hansen P. Storz G. (2013). Dual function of the McaS small RNA in controlling biofilm formation. *Genes Dev.* 27 1132–1145 10.1101/gad.214734.113 23666921 PMC3672647

[B58] Jutras B. L. Chenail A. M. Carroll D. W. Miller M. C. Zhu H. Bowman A. (2013a). Bpur, the Lyme disease spirochete’s PUR domain protein: identification as a transcriptional modulator and characterization of nucleic acid interactions. *J. Biol. Chem.* 288 26220–26234 10.1074/jbc.M113.491357 23846702 PMC3764826

[B59] Jutras B. L. Jones G. S. Verma A. Brown N. A. Antonicello A. D. Chenail A. M. (2013b). Posttranscriptional self-regulation by the Lyme disease bacterium’s BpuR DNA/RNA-binding protein. *J. Bacteriol.* 195 4915–4923 10.1128/JB.00819-13 23974034 PMC3807498

[B60] Kaberdin V. R. Singh D. Lin-Chao S. (2011). Composition and conservation of the mRNA-degrading machinery in bacteria. *J. Biomed. Sci*. 18 23 10.1186/1423-0127-18-23 PMC3071783 21418661

[B61] Kawamoto H. Koide Y. Morita T. Aiba H. (2006). Base-pairing requirement for RNA silencing by a bacterial small RNA and acceleration of duplex formation by Hfq. *Mol. Microbiol.* 61 1013–1022 10.1111/j.1365-2958.2006.05288.x 16859494

[B62] Kime L. Jourdan S. S. Stead J. A. Hidalgo-Sastre A. McDowall K. J. (2010). Rapid cleavage of RNA by RNase E in the absence of 5’ monophosphate stimulation. *Mol. Microbiol.* 76 590–604 10.1111/j.1365-2958.2009.06935.x 19889093 PMC2948425

[B63] Koleva R. I. Austin C. A. Kowaleski J. M. Neems D. S. Wang L. Vary C. P. H. (2006). Interactions of ribosomal protein S1 with DsrA and rpoS mRNA. *Biochem. Biophys. Res. Commun.* 348 662–668 10.1016/j.bbrc.2006.07.102 16890206

[B64] Komarova A. V. Tchufistova L. S. Dreyfus M. Boni I. V. (2005). AU-Rich sequences within 5’ untranslated leaders enhance translation and stabilize mRNA in *Escherichia coli*. *J. Bacteriol*. 187 1344–1349 10.1128/JB.187.4.1344 15687198 PMC545611

[B65] Kopaskie K. S. Ligtenberg K. G. Schneewind O. (2013). Translational regulation of *Yersinia enterocolitica* mRNA encoding a type III secretion substrate. *J. Biol. Chem.* 288 35478–35488 10.1074/jbc.M113.504811 24158443 PMC3853294

[B66] Kovach A. R. Hoff K. E. Canty J. T. Orans J. Brennan R. G. (2014). Recognition of U-rich RNA by Hfq from the Gram-positive pathogen *Listeria monocytogenes*. *RNA* 20 1548–1559 10.1261/rna.044032.113 25150227 PMC4174437

[B67] Kulkarni P. R. Cui X. Williams J. W. Stevens A. M. Kulkarni R. V. (2006). Prediction of CsrA-regulating small RNAs in bacteria and their experimental verification in *Vibrio fischeri*. *Nucleic Acids Res.* 34 3361–3369 10.1093/nar/gkl439 16822857 PMC1488887

[B68] Lawhon S. D. Frye J. G. Suyemoto M. Porwollik S. McClelland M. Altier C. (2003). Global regulation by CsrA in *Salmonella typhimurium*. *Mol. Microbiol.* 48 1633–1645 10.1046/j.1365-2958.2003.03535.x 12791144

[B69] Le Derout J. (2003). Hfq affects the length and the frequency of short oligo(A) tails at the 3’ end of *Escherichia coli* rpsO mRNAs. *Nucleic Acids Res.* 31 4017–4023 10.1093/nar/gkg456 12853618 PMC165971

[B70] Le Derout J. Boni I. V. Régnier P. Hajnsdorf E. (2010). Hfq affects mRNA levels independently of degradation. *BMC Mol. Biol.* 11:17 10.1186/1471-2199-11-17. PMC2834685 20167073

[B71] Link T. M. Valentin-Hansen P. Brennan R. G. (2009). Structure of *Escherichia coli* Hfq bound to polyriboadenylate RNA. *Proc. Natl. Acad. Sci. U.S.A.* 106 19292–19297 10.1073/pnas.0908744106 19889981 PMC2773200

[B72] Liu J. M. Camilli A. (2010). A broadening world of bacterial small RNAs. *Curr. Opin. Microbiol.* 13 18–23 10.1016/j.mib.2009.11.004 20022798 PMC2822007

[B73] Liu M. Y. Gui G. Wei B. Preston J. F. Oakford L. Yuksel U. (1997). The RNA molecule CsrB binds to the global regulatory protein CsrA and antagonizes its activity in *Escherichia coli*. *J. Biol. Chem.* 272 17502–17510 10.1074/jbc.272.28.17502 9211896

[B74] Liu M. Y. Yang H. Romeo T. (1995). The product of the pleiotropic *Escherichia coli* gene csrA modulates glycogen biosynthesis via effects on mRNA stability. *J. Bacteriol.* 177 2663–2672. 7751274 10.1128/jb.177.10.2663-2672.1995 PMC176935

[B75] Liu Y. Wu N. Dong J. Gao Y. Zhang X. Mu C. (2010). Hfq is a global regulator that controls the pathogenicity of *Staphylococcus aureus*. *PLoS ONE* 5:13069 10.1371/journal.pone.0013069 PMC2947504 20927372

[B76] Lorenz C. Gesell T. Zimmermann B. Schoeberl U. Bilusic I. Rajkowitsch L. (2010). Genomic SELEX for Hfq-binding RNAs identifies genomic aptamers predominantly in antisense transcripts. *Nucleic Acids Res.* 38 3794–3808 10.1093/nar/gkq032 20348540 PMC2887942

[B77] Lu Y. Turner R. Switzer R. (1996). Function of RNA secondary structures in transcriptional attenuation of the *Bacillus subtilis* pyr operon. *Proc. Natl. Acad. Sci. U.S.A.* 93 14462–14467 10.1073/pnas.93.25.14462 8962074 PMC26155

[B78] Mackay J. P. Font J. Segal D. J. (2011). The prospects for designer single-stranded RNA-binding proteins. *Nat. Struct. Mol. Biol.* 18 256–261 10.1038/nsmb.2005 21358629

[B79] Mackie G. A. (1998). Ribonuclease E is a 5’-end-dependent endonuclease. *Nature* 395 720–723 10.1038/27246 9790196

[B80] Mackie G. A. (2013). RNase E: at the interface of bacterial RNA processing and decay. *Nat. Rev. Microbiol.* 11 45–57 10.1038/nrmicro2930 23241849

[B81] Mahadevan S. Wright A. (1987). A bacterial gene involved in transcription antitermination: regulation at a rho-independent terminator in the bgl operon of E. *coli. Cell* 50 485–494 10.1016/0092-8674(87)90502-2 3301003

[B82] Mallory A. Vaucheret H. (2010). Form, function, and regulation of ARGONAUTE proteins. *Plant Cell* 22 3879–3889 10.1105/tpc.110.080671 21183704 PMC3027166

[B83] Marden J. N. Diaz M. R. Walton W. G. Gode C. J. Betts L. Urbanowski M. L. (2013). An unusual CsrA family member operates in series with RsmA to amplify posttranscriptional responses in *Pseudomonas aeruginosa*. *Proc. Natl. Acad. Sci. U.S.A.* 110 15055–15060 10.1073/pnas.1307217110 23980177 PMC3773774

[B84] Massé E. Escorcia F. E. Gottesman S. (2003). Coupled degradation of a small regulatory RNA and its mRNA targets in *Escherichia coli*. *Genes Dev*. 19 2374–2383 10.1101/gad.1127103 12975324 PMC218075

[B85] Merino E. Babitzke P. Yanofsky C. Merino E. Babitzke P. Yanofsky C. (1995). trp RNA-binding attenuation protein (TRAP)-trp leader RNA interactions mediate translational as well as transcriptional regulation of the *Bacillus subtilis* trp operon. *J. Bacteriol.* 177 6362–6370. 7592410 10.1128/jb.177.22.6362-6370.1995 PMC177485

[B86] Mikulecky P. J. Kaw M. K. Brescia C. C. Takach J. C. Darren D. Feig A. L. (2004). *Escherichia coli* Hfq has distinct interaction surfaces for DsrA, rpoS and poly(A) RNAs. *Nat. Struct. Mol. Biol.* 11 1206–1214 10.1038/nsmb858 PMC3071270 15531892

[B87] Milojevic T. Grishkovskaya I. Sonnleitner E. Djinovic-Carugo K. Bläsi U. (2013). The *Pseudomonas aeruginosa* catabolite repression control protein Crc is devoid of RNA binding activity. *PLoS ONE* 8:e64609 10.1371/journal.pone.0064609 PMC3662782 23717639

[B88] Mitobe J. Yanagihara I. Ohnishi K. Yamamoto S. Ohnishi M. Ishihama A. (2011). RodZ regulates the post-transcriptional processing of the *Shigella sonnei* type III secretion system. *EMBO Rep.* 12 911–916 10.1038/embor.2011.132 21779005 PMC3166458

[B89] Mohanty B. K. Maples V. F. Kushner S. R. (2004). The Sm-like protein Hfq regulates polyadenylation dependent mRNA decay in *Escherichia coli*. *Mol. Microbiol.* 54 905–920 10.1111/j.1365-2958.2004.04337.x 15522076

[B90] Moll I. Afonyushkin T. Vytvytska O. Kaberdin V. R. (2003). Coincident Hfq binding and RNase E cleavage sites on mRNA and small regulatory RNAs. *RNA* 11 1308–1314 10.1261/rna.5850703 14561880 PMC1287052

[B91] Møller T. Franch T. Højrup P. Keene D. R. Ba H. P. Brennan R. G. (2002). Hfq: a bacterial sm-like protein that mediates RNA-RNA interaction. *Mol. Cell* 9 23–30 10.1016/S1097-2765(01)00436-1 11804583

[B92] Moreno R. Hernández-Arranz S. La Rosa R. Yuste L. Madhushani A. Shingler V. (2014). The Crc and Hfq proteins of *Pseudomonas putida* cooperate in catabolite repression and formation of ribonucleic acid complexes with specific target motifs. *Environ. Microbiol.* 17 105–118 10.1111/1462-2920.12499 24803210

[B93] Moreno R. Marzi S. Romby P. Rojo F. (2009). The Crc global regulator binds to an unpaired A-rich motif at the *Pseudomonas putida* alkS mRNA coding sequence and inhibits translation initiation. *Nucleic Acids Res.* 37 7678–7690 10.1093/nar/gkp825 19825982 PMC2794181

[B94] Morita T. Maki K. Aiba H. (2005). RNase E-based ribonucleoprotein complexes: mechanical basis of mRNA destabilization mediated by bacterial noncoding RNAs. *Genes Dev*. 19 2176–2186 10.1101/gad.1330405.1996 16166379 PMC1221888

[B95] Morris E. R. Hall G. Li C. Heeb S. Kulkarni R. V. Lovelock L. (2013). Structural rearrangement in an RsmA/CsrA Ortholog of *pseudomonas aeruginosa* creates a dimeric RNA-binding protein, RsmN. *Structure* 21 1659–1671 10.1016/j.str.2013.07.007 23954502 PMC3791407

[B96] Muﬄer A. Fischer D. Hengge-Aronis R. (1996). The RNA-binding protein HF-I, known as a host factor for phage Qbeta RNA replication, is essential for rpoS translation in *Escherichia coli*. *Genes Dev.* 10 1143–1151 10.1101/gad.10.9.1143 8654929

[B97] Mukherjee S. Yakhnin H. Kysela D. Sokoloski J. Babitzke P. Kearns D. B. (2011). CsrA-FliW interaction governs flagellin homeostasis and a checkpoint on flagellar morphogenesis in *Bacillus subtilis*. *Mol. Microbiol.* 82 447–461 10.1111/j.1365-2958.2011.07822.x 21895793 PMC3192257

[B98] Muto Y. Oubridge C. Nagai K. (2000). RNA-binding proteins: trapping RNA bases. *Curr. Biol.* 10 19–21 10.1016/S0960-9822(99)00250-X 10660288

[B99] Osborne J. Djapgne L. Tran B. Q. Goo Y. A. Oglesby-Sherrouse A. G. (2014). A method for in vivo identification of bacterial small RNA-binding proteins. *Microbiologyopen* 3 950–960 10.1002/mbo3.220 25351924 PMC4263517

[B100] Pandey P, S. Winkler J. A. Li H. Camacho D. M. Collins J. J. Walker G. C. (2014). Central role for RNase YbeY in Hfq-dependent and Hfq-independent small-RNA regulation in bacteria. *BMC Genomics* 15:121 10.1186/1471-2164-15-121 PMC3933206 24511998

[B101] Pandey S. P. Minesinger B. K. Kumar J. Walker G. C. (2011). A highly conserved protein of unknown function in Sinorhizobium meliloti affects sRNA regulation similar to Hfq. *Nucleic Acids Res.* 39 4691–4708 10.1093/nar/gkr060 21325267 PMC3113577

[B102] Patterson-Fortin L. M. Vakulskas C. A. Yakhnin H. Babitzke P. Romeo T. (2013). Dual posttranscriptional regulation via a cofactor-responsive mRNA leader. *J. Mol. Biol.* 425 3662–3677 10.1016/j.jmb.2012.12.010 23274138 PMC3710303

[B103] Perez-Rueda E. Martinez-Nuñez M. A. (2012). The repertoire of DNA-binding transcription factors in prokaryotes: functional and evolutionary lessons. *Sci. Prog.* 95 315–329 10.3184/003685012X13420097673409 23094327 PMC10365527

[B104] Phadtare S. Inouye M. Severinov K. (2002). The nucleic acid melting activity of *Escherichia coli* CspE is critical for transcription antitermination and cold acclimation of cells. *J. Biol. Chem.* 277 7239–7245 10.1074/jbc.M111496200 11756430

[B105] Picard F. Dressaire C. Girbal L. Cocaign-Bousquet M. (2009). Examination of post-transcriptional regulations in prokaryotes by integrative biology. *C. R. Biol.* 332 958–973 10.1016/j.crvi.2009.09.005 19909919

[B106] Rabhi M. Espéli O. Schwartz A. Cayrol B. Rahmouni A. R. Arluison V. (2011). The Sm-like RNA chaperone Hfq mediates transcription antitermination at Rho-dependent terminators. *EMBO J.* 30 2805–2816 10.1038/emboj.2011.192 21673658 PMC3160251

[B107] Ramesh A. DebRoy S. Goodson J. R. Fox K. A. Faz H. Garsin D. A. (2012). The mechanism for RNA recognition by ANTAR regulators of gene expression. *PLoS Genet.* 8:e1002666 10.1371/journal.pgen.1002666 PMC3369931 22685413

[B108] Rasmussen A. A. Eriksen M. Gilany K. Udesen C. Franch T. Petersen C. (2005). Regulation of ompA mRNA stability: the role of a small regulatory RNA in growth phase-dependent control. *Mol. Microbiol.* 58 1421–1429 10.1111/j.1365-2958.2005.04911.x 16313626

[B109] Régnier P. Hajnsdorf E. (2013). The interplay of Hfq, poly(A) polymerase I and exoribonucleases at the 3’ ends of RNAs resulting from Rho-independent termination: a tentative model. *RNA Biol.* 10 602–609 10.4161/rna.23664 23392248 PMC3710367

[B110] Reimmann C. Valverde C. Kay E. Haas D. (2005). Posttranscriptional repression of GacS / GacA-controlled genes by the RNA-binding protein RsmE acting together with RsmA in the biocontrol strain *Pseudomonas fluorescens* CHA0. *J. Bacteriol.* 187 276–285 10.1128/JB.187.1.276 15601712 PMC538806

[B111] Rieder R. Reinhardt R. Sharma C. Vogel J. (2012). Experimental tools to identify RNA-protein interactions in *Helicobacter pylori*. *RNA Biol.* 9 520–531 10.4161/rna.20331 22546936

[B112] Romeo T. Vakulskas C. A. Babitzke P. (2013). Post-transcriptional regulation on a global scale: form and function of Csr/Rsm systems. *Environ. Microbiol.* 15 313–324 10.1111/j.1462-2920.2012.02794.x 22672726 PMC3443267

[B113] Rutberg B. (1997). MicroReview antitermination of transcription of catabolic operons. *Mol. Microbiol.* 23 413–421 10.1046/j.1365-2958.1997.d01-1867.x 9044276

[B114] Said N. Rieder R. Hurwitz R. Deckert J. Urlaub H. Vogel J. (2009). In vivo expression and purification of aptamer-tagged small RNA regulators. *Nucleic Acids Res*. 37 e133. 10.1093/nar/gkp719 PMC2777422 19726584

[B115] Salvail H. Caron M.-P. Bélanger J. Massé E. (2013). Antagonistic functions between the RNA chaperone Hfq and an sRNA regulate sensitivity to the antibiotic colicin. *EMBO J.* 32 2764–2778 10.1038/emboj.2013.205 24065131 PMC3801439

[B116] Santangelo T. J. Artsimovitch I. (2011). Termination and antitermination: RNA polymerase runs a stop sign. *Nat. Rev. Microbiol.* 9 319–329 10.1038/nrmicro2560 21478900 PMC3125153

[B117] Saramago M. Bárria C. Dos Santos R. F. Silva I. J. Pobre V. Domingues S. (2014). The role of RNases in the regulation of small RNAs. *Curr. Opin. Microbiol.* 18 105–115 10.1016/j.mib.2014.02.009 24704578

[B118] Sarsero J. P. Merino E. Yanofsky C. (2000). A *Bacillus subtilis* gene of previously unknown function, yhaG, is translationally regulated by tryptophan-activated TRAP and appears to be involved in tryptophan transport. *J. Bacteriol.* 182 2329–2331 10.1128/JB.182.8.2329-2331.2000 10735881 PMC111287

[B119] Sauer E. (2013). Structure and RNA-binding properties of the bacterial LSm protein Hfq. *RNA Biol.* 10 610–618 10.4161/rna.24201 23535768 PMC3710368

[B120] Sauer E. Schmidt S. Weichenrieder O. (2012). Small RNA binding to the lateral surface of Hfq hexamers and structural rearrangements upon mRNA target recognition. *Proc. Natl. Acad. Sci. U.S.A.* 109 9396–9401 10.1073/pnas.1202521109 22645344 PMC3386104

[B121] Schiano C. A. Lathem W. W. (2012). Post-transcriptional regulation of gene expression in *Yersinia* species. *Front. Cell. Infect. Microbiol.* 2:129 10.3389/fcimb.2012.00129 PMC3493969 23162797

[B122] Schnetz K. Rak B. (1988). Regulation of the bgl operon of *Escherichia coli* by transcriptional antitermination. *EMBO J*. 7 3271–3277. 2846278 10.1002/j.1460-2075.1988.tb03194.x PMC454751

[B123] Schumacher M. A. Pearson R. F. Møller T. Valentin-Hansen P. Brennan R. G. (2002). Structures of the pleiotropic translational regulator Hfq and an Hfq-RNA complex: a bacterial Sm-like protein. *EMBO J.* 21 3546–3556 10.1093/emboj/cdf322 12093755 PMC126077

[B124] Shahbabian K. Jamalli A. Zig L. Putzer H. (2009). RNase Y, a novel endoribonuclease, initiates riboswitch turnover in *Bacillus subtilis*. *EMBO J.* 28 3523–3533 10.1038/emboj.2009.283 19779461 PMC2782095

[B125] Sheidy D. T. Zielke R. A. (2013). Analysis and expansion of the role of the *Escherichia coli* protein ProQ. *PLoS ONE* 8:e79656 10.1371/journal.pone.0079656 PMC3808355 24205389

[B126] Shine J. Dalgarno L. (1974). The 3’-terminal sequence of *Escherichia coli* 16S ribosomal RNA: complementarity to nonsense triplets and ribosome binding sites. *Proc. Natl. Acad. Sci. U.S.A.* 71 1342–1346 10.1073/pnas.71.4.1342 4598299 PMC388224

[B127] Smaldone G. T. Antelmann H. Gaballa A. Helmann J. D. (2012). The FsrA sRNA and FbpB protein mediate the iron-dependent induction of the *Bacillus subtilis* lutABC iron-sulfur-containing oxidases. *J. Bacteriol.* 194 2586–2593 10.1128/JB.05567-11 22427629 PMC3347220

[B128] Snyder D. Lary J. Chen Y. Gollnick P. Cole J. L. (2004). Interaction of the trp RNA-binding attenuation protein (TRAP) with anti-TRAP. *J. Mol. Biol.* 338 669–682 10.1016/j.jmb.2004.03.030 15099736

[B129] Sobrero P. Valverde C. (2012). The bacterial protein Hfq: much more than a mere RNA-binding factor. *Crit. Rev. Microbiol.* 38 276–299 10.3109/1040841X.2012.664540 22435753

[B130] Sonnleitner E. Abdou L. Haas D. (2009). Small RNA as global regulator of carbon catabolite repression in *Pseudomonas aeruginosa*. *Proc. Natl. Acad. Sci. U.S.A.* 106 21866–21871 10.1073/pnas.pnas.0910308106 20080802 PMC2799872

[B131] Sonnleitner E. Bläsi U. (2014). Regulation of Hfq by the RNA CrcZ in *Pseudomonas aeruginosa* carbon catabolite repression. *PLoS Genet.* 10:e1004440 10.1371/journal.pgen.1004440 PMC4063720 24945892

[B132] Soper T. J. Doxzen K. Woodson S. A. (2011). Major role for mRNA binding and restructuring in sRNA recruitment by Hfq. *RNA* 17 1544–1550 10.1261/rna.2767211 21705431 PMC3153977

[B133] Sorger-Domenigg T. Sonnleitner E. Kaberdin V. R. Bläsi U. (2007). Distinct and overlapping binding sites of *Pseudomonas aeruginosa* Hfq and RsmA proteins on the non-coding RNA RsmY. *Biochem. Biophys. Res. Commun.* 352 769–773 10.1016/j.bbrc.2006.11.084 17141182

[B134] Sterzenbach T. Nguyen K. T. Nuccio S.-P. Winter M. G. Vakulskas C. A. Clegg S. (2013). A novel CsrA titration mechanism regulates fimbrial gene expression in *Salmonella typhimurium*. *EMBO J.* 32 2872–2883 10.1038/emboj.2013.206 24056837 PMC3817462

[B135] Storz G. Vogel J. Wassarman K. M. (2011). Regulation by small RNAs in bacteria: expanding frontiers. *Mol. Cell* 43 880–891 10.1016/j.molcel.2011.08.022 21925377 PMC3176440

[B136] Stülke J. (2002). Control of transcription termination in bacteria by RNA-binding proteins that modulate RNA structures. *Arch. Microbiol.* 177 433–440 10.1007/s00203-002-0407-5 12029388

[B137] Subramanian A. R. (1983). Structure and functions of ribosomal protein S1. *Prog. Nucleic Acid Res. Mol. Biol.* 28 101–142 10.1016/S0079-6603(08)60085-9 6348874

[B138] Suzuki K. Babitzke P. Kushner S. R. Romeo T. (2006). Identification of a novel regulatory protein ( CsrD ) that targets the global regulatory RNAs CsrB and CsrC for degradation by RNase E. *Genes Dev*. 20 2605–2617 10.1101/gad.1461606 16980588 PMC1578682

[B139] Tortosa P. Lindner C. Saier M. H. Reizer J. Le Coq D. (1997). Multiple Phosphorylation of SacY, a *Bacillus subtilis* transcriptional antiterminator negatively controlled by the phosphotransferase system. *J. Biol. Chem.* 272 17230–17237 10.1074/jbc.272.27.17230 9202047

[B140] Tsai B. P. Wang X. Huang L. Waterman M. L. (2011). Quantitative profiling of in vivo-assembled RNA-protein complexes using a novel integrated proteomic approach. *Mol. Cell Proteomics* 10 M110.007385. 10.1074/mcp.M110.007385 PMC3069349 21285413

[B141] Tsui H. C. Leung H. C. Winkler M. E. (1994). Characterization of broadly pleiotropic phenotypes caused by an hfq insertion mutation in *Escherichia coli* K–12. *Mol. Microbiol.* 13 35–49 10.1111/j.1365-2958.1994.tb00400.x 7984093

[B142] Vecerek B. Moll I. Bläsi U. (2005). Translational autocontrol of the *Escherichia coli* hfq RNA chaperone gene. *RNA* 11 976–984 10.1261/rna.2360205 15872186 PMC1370782

[B143] Vercruysse M. Köhrer C. Davies B. W. Arnold M. F. F. Mekalanos J. J. RajBhandary U. L. (2014). The highly conserved bacterial RNase YbeY is essential in vibrio cholerae, playing a critical role in virulence, stress regulation, and RNA processing. *PLoS Pathog.* 10:e1004175 10.1371/journal.ppat.1004175 PMC4047096 24901994

[B144] Vogel J. Luisi B. F. (2011). Hfq and its constellation of RNA. *Nat. Rev. Microbiol.* 9 578–589 10.1038/nrmicro2615 21760622 PMC4615618

[B145] Vytvytska O. Moll I. Kaberdin V. R. Von Gabain A. Bläsi U. (2000). binding Hfq ( HF1 ) stimulates ompA mRNA decay by interfering with ribosome binding. *Genes Dev*. 14 1109–1118 10.1101/gad.14.9.1109 10809669 PMC316587

[B146] Wang M.-C. Chien H.-F. Tsai Y.-L. Liu M.-C. Liaw S.-J. (2014). The RNA chaperone Hfq is involved in stress tolerance and virulence in uropathogenic *Proteus mirabilis*. *PLoS ONE* 9:e85626 10.1371/journal.pone.0085626 PMC3893223 24454905

[B147] Wang X. Dubey A. K. Suzuki K. Baker C. S. Babitzke P. Romeo T. (2005). CsrA post-transcriptionally represses pgaABCD, responsible for synthesis of a biofilm polysaccharide adhesin of *Escherichia coli*. *Mol. Microbiol.* 56 1648–1663 10.1111/j.1365-2958.2005.04648.x 15916613

[B148] Weilbacher T. Suzuki K. Dubey A. K. Wang X. Gudapaty S. Morozov I. (2003). A novel sRNA component of the carbon storage regulatory system of *Escherichia coli*. *Mol. Microbiol.* 48 657–670 10.1046/j.1365-2958.2003.03459.x 12694612

[B149] Windbichler N. von Pelchrzim F. Mayer O. Csaszar E. Schroeder R. (2008). Isolation of small RNA-binding proteins from E. *coli:* evidence for frequent interaction of RNAs with RNA polymerase. *RNA Biol.* 5 30–40 10.4161/rna.5.1.5694 18388495

[B150] Yakhnin A. V Baker C. S. Vakulskas C. A. Yakhnin H. Berezin I. Romeo T. (2014). CsrA activates flhDC expression by protecting flhDC mRNA from RNase E-mediated cleavage. *Mol. Cell* 87 851–866 10.1111/mmi.12136.CsrA PMC3567230 23305111

[B151] Yakhnin H. Baker C. S. Berezin I. Evangelista M. A. Rassin A. Romeo T. (2011a). CsrA represses translation of sdiA, which encodes the N-acylhomoserine-L-lactone receptor of *Escherichia coli*, by binding exclusively within the coding region of sdiA mRNA. *J. Bacteriol.* 193 6162–6170 10.1128/JB.05975-11 21908661 PMC3209218

[B152] Yakhnin H. Yakhnin A. V. Baker C. S. Sineva E. Berezin I. Romeo T. (2011b). Complex regulation of the global regulatory gene csrA: CsrA-mediated translational repression, transcription from five promoters by Esigma70 andEsigmaS, and indirect transcriptional activation by CsrA. *Mol. Microbiol.* 81 689–704 10.1111/j.1365-2958.2011.07723.x 21696456 PMC3189700

[B153] Yakhnin H. Zhang H. Yakhnin A. V. Babitzke P. (2004). The trp RNA-binding attenuation protein of *Bacillus subtilis* regulates translation of the tryptophan transport gene trpP ( yhaG ) by blocking ribosome binding. *J. Bacteriol.* 186 278–286 10.1128/JB.186.2.278 14702295 PMC305772

[B154] Yang M. de Saizieu A. van Loon A. P. Gollnick P. (1995). Translation of trpG in *Bacillus subtilis* is regulated by the trp RNA-binding attenuation protein (TRAP). *J. Bacteriol.* 177 4272–4278. 7543470 10.1128/jb.177.15.4272-4278.1995 PMC177173

[B155] Zha D. Xu L. Zhang H. Yan Y. (2014). The two-component GacS-GacA System activates lipA translation by RsmE but not RsmA in *Pseudomonas* protegens Pf-5. *Appl. Environ. Microbiol.* 80 6627–6637 10.1128/AEM.02184-14 PMC4249036 25128345

[B156] Zhang A. Wassarman K. M. Ortega J. Steven A. C. Storz G. (2002). The Sm-like Hfq protein increases OxyS RNA interaction with target mRNAs. *Mol. Cell* 9 11–22 10.1016/S1097-2765(01)00437-3 11804582

